# Inverse Design of Tetracene Polymorphs with Enhanced Singlet Fission Performance by Property-Based Genetic Algorithm Optimization

**DOI:** 10.1021/acs.chemmater.2c03444

**Published:** 2023-01-21

**Authors:** Rithwik Tom, Siyu Gao, Yi Yang, Kaiji Zhao, Imanuel Bier, Eric A. Buchanan, Alexandr Zaykov, Zdeněk Havlas, Josef Michl, Noa Marom

**Affiliations:** † Department of Physics, Carnegie Mellon University, Pittsburgh, Pennsylvania15213, United States; ‡ Department of Materials Science and Engineering, Carnegie Mellon University, Pittsburgh, Pennsylvania15213, United States; § Department of Chemistry, University of Colorado, Boulder, Colorado80309, United States; ∥ Institute of Organic Chemistry and Biochemistry, Czech Academy of Sciences, 16610Prague 6, Czech Republic; ⊥ Department of Physical Chemistry, University of Chemistry and Technology, 166 28Prague 6, Czech Republic; # Department of Chemistry, Carnegie Mellon University, Pittsburgh, Pennsylvania15213, United States

## Abstract

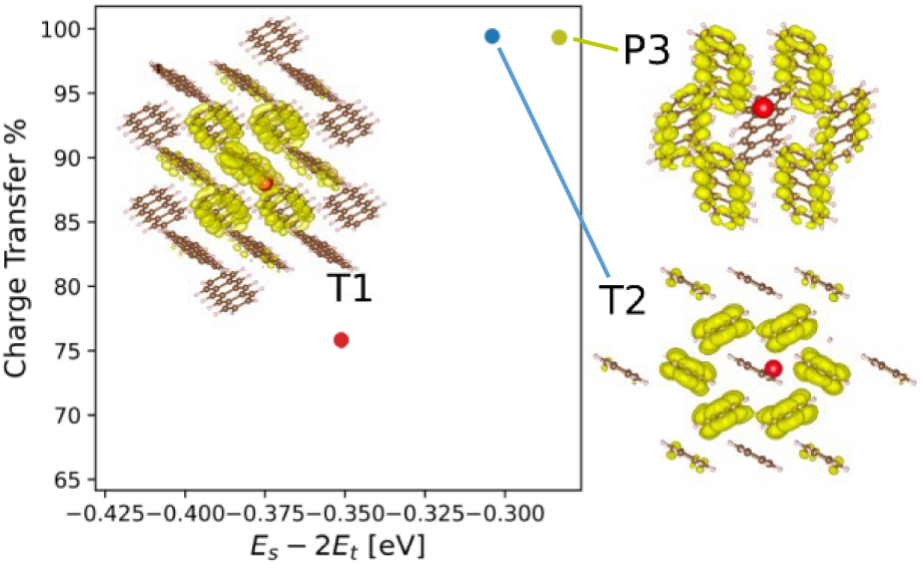

The efficiency of solar cells may be improved by using singlet fission (SF), in which one singlet exciton splits into two triplet excitons. SF occurs in molecular crystals. A molecule may crystallize in more than one form, a phenomenon known as polymorphism. Crystal structure may affect SF performance. In the common form of tetracene, SF is experimentally known to be slightly endoergic. A second, metastable polymorph of tetracene has been found to exhibit better SF performance. Here, we conduct inverse design of the crystal packing of tetracene using a genetic algorithm (GA) with a fitness function tailored to simultaneously optimize the SF rate and the lattice energy. The property-based GA successfully generates more structures predicted to have higher SF rates and provides insight into packing motifs associated with improved SF performance. We find a putative polymorph predicted to have superior SF performance to the two forms of tetracene, whose structures have been determined experimentally. The putative structure has a lattice energy within 1.5 kJ/mol of the most stable common form of tetracene.

## Introduction

The maximum efficiency of a single-junction solar cell is given by the Shockley–Queisser limit,^1^ which assumes that one photon is converted into one charge carrier. Under ambient conditions, the resulting conversion efficiency is approximately 33%. One of the main reasons for performance degradation is the energy loss when high-energy excitons relax to the band edge, wasting the excess energy of high-energy photons as heat. This may be overcome by multiexciton generation methods in which a high-energy photon can be split into lower-energy excitons that are efficiently harvested.^2^ Thus, the limit of efficiency of a solar cell may be enhanced to nearly 47%.^3^

Singlet fission (SF) is a multiexciton generation phenomenon observed in molecular crystals, where a singlet-state exciton is converted into two triplet-state excitons.^4−6^ SF was discovered as early as 1965 in crystalline anthracene.^7^ To date, certain materials have been observed to undergo SF in the solid state, including acenes,^4,5,8−11^ rylenes,^12−15^ diphenylisobenzofuran,^16,17^ carotenoids,^18−20^ and thiophenes.^21−24^ For a material to undergo SF, the adiabatic singlet exciton energy should be greater than twice the adiabatic triplet exciton energy. A singlet exciton with more electron delocalization on neighboring molecules can potentially improve the coupling between singlet and triplet states.^25−28^ Thus, a higher degree of charge transfer character (%CT) in the lowest singlet excited state is considered beneficial to SF.^4,6,29^ In addition to efficient SF, practical applications in solar cells require several desirable properties such as photostability, conductivity, and exciton diffusibility.^29,30^ This has motivated the search for new classes of SF materials that are commercially viable. Recently, several experimental^31,32^ and computational^28,33−38^ studies have focused on identifying new candidate materials for SF.

**Figure 1:**
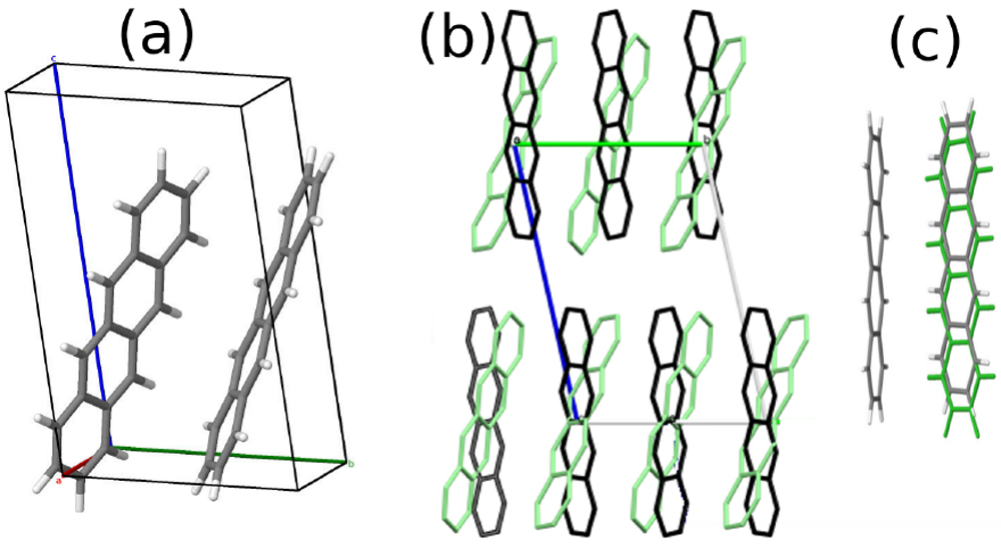
Two experimentally observed forms of tetracene: (a) the common T1 polymorph^54^ and (b) the second form, T2,^55^ viewed along the *c*-axis with the molecules colored green and the T1 molecules colored black, overlaid using the Cambridge Crystallographic Data Centre (CCDC) packing similarity tool.^59^ (c) Overlay of the molecules of the T1 form in gray and the T2 form in green.

The electronic and optical properties of a molecular crystal depend on how the molecules are packed and the resulting electronic coupling between them. Polymorphs, i.e., different crystal structures of the same molecule, can have significantly different properties.^35,39,40^ Understanding the effect of crystal packing on singlet fission (SF) has been of recent interest.^5,6,35,41−45^ Tetracene, one of the earliest known SF materials, has been reported to exhibit polymorphism.^46−48^ The SF in the common form of tetracene has been found to be slightly endoergic.^49^ This means that modifying the crystal packing may potentially shift the singlet and triplet excitation energies to make SF more or less favorable. Two unique crystal structures of tetracene are available in the Cambridge Structural Database (CSD).^46,50−53^ The T1 crystal structure, shown in Figure 1a, is the commonly occurring bulk phase of tetracene.^54^ T2, shown in Figure 1b, is a polymorph found predominantly in thin films.^55,56^ Both forms of tetracene contain two molecules per unit cell. As shown in panels b and c of Figure 1, the molecule pair of the T1 crystal structure (gray) is slipped relative to that of T2 (green). A detailed account of the differences in the structure of these polymorphs and their experimental synthesis and characterization can be found in ref (55). SF in a thin film form of tetracene was experimentally found to be significantly faster than in the stable T1 polymorph.^57^ The crystal structure therein was not fully solved to determine the atomic positions; however, it is assumed by the authors to be T2 on the basis of the lattice parameters. The transfer of triplet excitons was also found to be more efficient for T2, potentially making it a better candidate than T1 for SF-sensitized silicon cells.^58^ This demonstrates that the specific crystal packing of tetracene is crucial for its SF performance.

In addition to the thermodynamic driving force, which results from the energy balance between the singlet and triplet excited states, kinetic considerations affect the SF performance of a molecular crystal. To elucidate the effect of crystal packing on SF rates, Michl and Havlas developed a dimer-based model,^60^ implemented in the “Simple” code.^61^ Simple was not designed to predict absolute SF rates but to identify geometrical factors affecting SF for a given material. Thus, Simple may be helpful in the identification of optimal dimers for a specific molecule. The Simple model has been applied to dimers of ethylene,^61^ tetracene,^62^ 1,3-diphenylisobenzofuran,^43^ and other molecules.^63,64^ The results of these studies suggest that the SF rate is sensitive to slight changes in geometry, validating experimental observations. These studies have analyzed millions of chromophore orientations within a dimer and have provided useful guidance for optimal packing. However, the dimers found to be optimal are not those seen in the experimentally observed crystal structures. In particular, the dimer extracted from the T1 form of tetracene is close to only the 10th most optimal dimer found by Simple.^62^ It is thus challenging to realize a crystal with optimal dimer geometry for SF in practice. To find molecular packing arrangements that are experimentally synthesizable, it is important to consider the space of potential polymorphs, i.e., the local minima of the potential energy surface (PES) that are within a range of approximately 4 kJ/mol^65^ of the global minimum. For this purpose, tools for molecular crystal structure prediction (CSP) can be utilized.

CSP is the fundamental problem of predicting the crystal structure of a molecule using computer simulations.^66−69^ CSP is a notoriously challenging problem because it requires searching a high-dimensional space with high accuracy.^70,71^ A typical CSP workflow starts by generating putative crystal structures using random or quasi-random methods to adequately sample the configurational space.^72−75^ A variety of global optimization algorithms are used to explore the PES.^68,69,76,77^ These optimization schemes are coupled with hierarchical energy evaluations and geometry relaxations that employ increasingly more accurate and computationally expensive methods for reducing the number of candidates to be considered further. Dispersion-inclusive density functional theory (DFT) has become the method of choice for ranking candidate structures.^71,78^ The effect of temperature on ranking can be accounted for by applying vibrational corrections via the harmonic or quasi-harmonic approximation methods.^79,80^ Recently, CSP has been used in conjunction with experiments to discover potential polymorphs.^81,82^

While traditional CSP is focused on searching for the structures with the lowest energy, we are interested in structures with optimal SF performance. The search for a structure with a target property is known as inverse design.^83,84^ To this end, we use a property-based genetic algorithm (GA). A GA generates new offspring by performing crossover and mutation operations on the structural genes of parent structures. Structures are selected for mating on the basis of a fitness function, which assigns a higher probability of selection to fitter structures. This propagates desirable features in the population. The cycle of fitness evaluation, selection, and mating repeats until no better structures are found. For CSP, structures with lower energies (i.e., higher stability) have a higher fitness. An advantage of a GA over other methods is that the fitness function can be formulated to optimize any property of interest. GAs with tailor-made fitness functions have been used, for example, to optimize the electronic,^85^ optical,^86^ and excitonic^87^ properties of materials, as well as transport properties of organic semiconductor molecules.^88^ Unlike the global minimum search of traditional CSP, a property-based GA does not necessarily yield one optimum but a set of solutions with the target property and stability within an acceptable range. Once putative structures predicted to possess desirable properties are found, experimental synthesis may be pursued. It is possible to synthesize metastable molecular crystal polymorphs by a variety of experimental techniques.^89^ For example, changing the solvent and crystallization conditions (temperature, pressure, etc.),^90−92^ tailor-made additives,^93,94^ solution shearing,^95,96^ epitaxial templating,^97^ and nanoscale confinement^98−101^ can produce different polymorphs.

We have developed the GAtor GA code for molecular crystal structure prediction.^102−104^ To perform inverse design of crystal structures with enhanced SF performance, we have implemented in GAtor a property-based fitness function, tailored to simultaneously minimize the energy and maximize the SF rate. SF rates are evaluated by using Simple for dimers extracted from the crystal structures because this is a descriptor that is computationally efficient to evaluate within the GA. Our inverse design strategy is then applied to tetracene because its known polymorphs have been experimentally observed to display significantly different SF performance. The SF+energy-based fitness function successfully biases the GA to generate structures predicted to have higher SF rates. The resulting structures provide insight into what packing motifs would be likely to lead to enhanced SF performance. The structures found within the polymorph energy range are further evaluated by using many-body perturbation theory. We identify a structure with a thermodynamic driving force for SF higher than those of both known forms of tetracene and a singlet exciton with a high degree of charge transfer character. Furthermore, the lattice energy of this structure is only 1.5 kJ/mol higher than that of the most stable T1 structure of tetracene, and therefore it is likely to be experimentally synthesizable.

## Methods

### Approach

#### Workflow Overview

**Figure 2:**
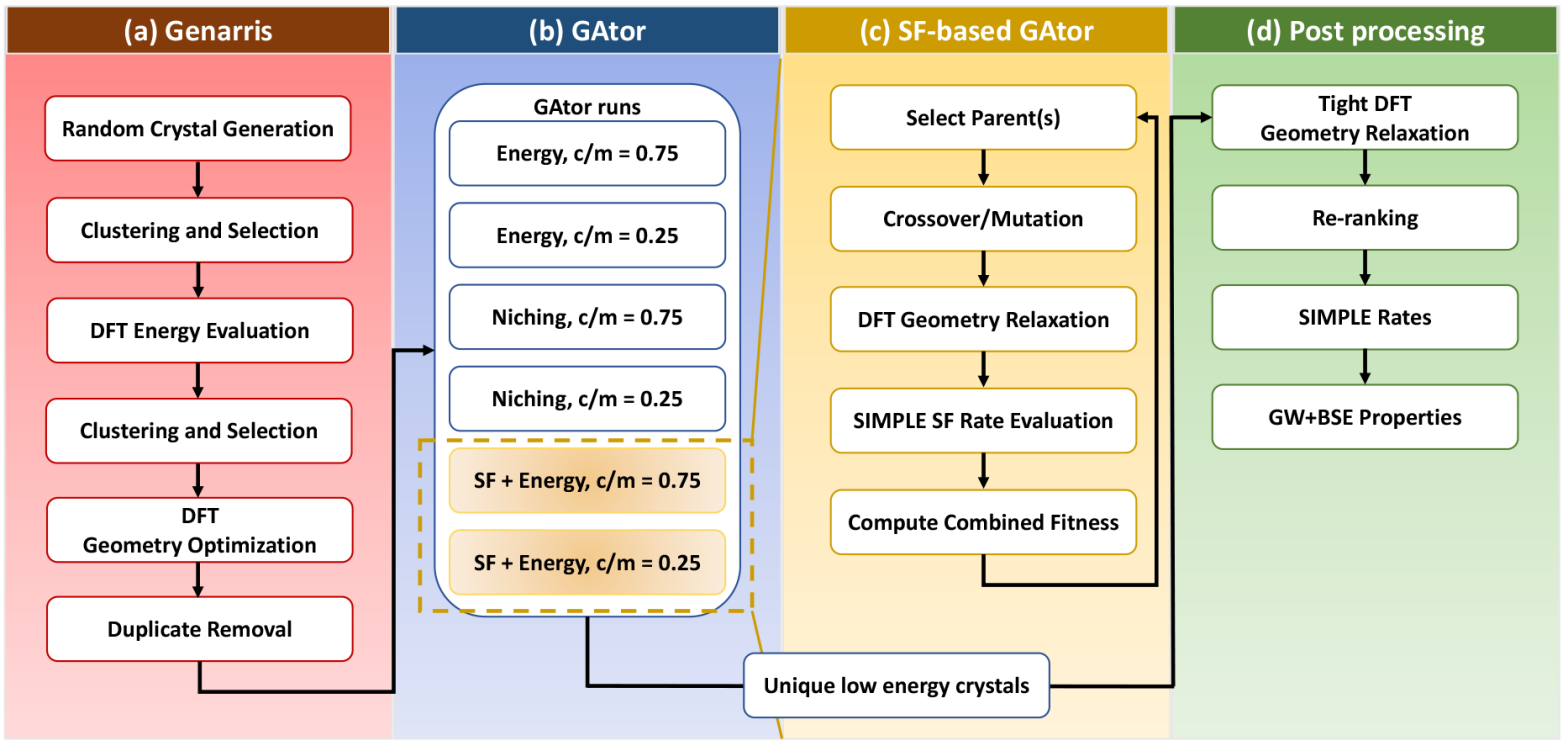
Overview of the inverse design workflow for finding putative molecular crystal polymorphs with improved SF performance. (a) Initial pool generation with Genarris. (b) GAtor runs are conducted using the traditional energy-based fitness function, the niching fitness function, and the SF+energy based fitness function. (c) Workflow of GAtor with the SF+energy based fitness function. (d) Postprocessing.

Figure 2 shows an overview of the inverse design workflow. The first step (panel a) is generating a pool of random structures using Genarris.^73^ Genarris randomly creates structures in all possible space groups consistent with the symmetry of the molecule and the requested number of molecules in the unit cell. Unit cells are generated with a distribution around a predicted volume.^105^ Fast, hierarchical structure checks are conducted to remove crystals with unphysically close intermolecular contact distances. An initial set of random structures undergo down-selection based on structural diversity and stability. Structural similarity is evaluated on the basis of a radial symmetry function (RSF) descriptor,^106^ and the affinity propagation (AP) machine learning algorithm is utilized for clustering.^107^ A final set of structures are fully relaxed with dispersion-inclusive DFT. This is used as the initial population to seed GAtor runs.

The next step (panel b) is conducting GA runs. GAtor has the following unique features. First, a massive parallelization scheme enables the efficient utilization of high-performance computing resources by running several GA replicas in parallel. The GA replicas pick parent crystals for mating, generate offspring, and relax them with DFT independently, and interact only through a shared population of structures.^87,102^ Second, several crossover and mutation schemes, designed for molecular crystals, are implemented in GAtor, which achieve a balance between exploration and exploitation by breaking or preserving space group symmetries. Third, in addition to the traditional energy-based fitness function, evolutionary niching has been implemented in GAtor to prevent the GA from being trapped in local minima and force it to explore structurally diverse basins.^103^ This helps overcome initial pool biases and selection biases, known as genetic drift.

To perform inverse design, we have implemented in GAtor a fitness function based on a combination of a high SF rate and a low energy. This adds to the GAtor workflow a step of calculating the SF rates of the structures in the population using Simple (panel c). Simple uses a dimer model to compute SF rates. The singlet exciton on the excited chromophore couples into a singlet biexciton, which then decomposes into two triplet excitons. Simple computes the rate of biexciton formation using the Fermi golden rule. The SF electronic matrix elements are computed on the basis of a frontier orbital model using only the highest occupied molecular orbital (HOMO) and the lowest unoccupied molecular orbital (LUMO) of the two molecules. To evaluate the SF rate of a molecular crystal using Simple, dimers are extracted from the crystal structure and the highest result obtained is taken as the SF rate of that structure.^33^ The computational efficiency of Simple is advantageous for fast evaluations within the GA. GAtor runs are conducted using the traditional energy-based fitness function, the niching fitness function, and the SF-based fitness function. With each fitness function, two runs are conducted with different crossover versus mutation rates. Our recommended best practice for CSP is to perform several GAtor runs with different settings.^102−104^

The best structures produced by GAtor undergo postprocessing (panel d). First, the structures are re-relaxed and re-ranked using increasingly accurate dispersion-inclusive DFT methods. Initially, the structures are relaxed and ranked using the computationally efficient Perdew–Burke–Ernzerhof (PBE)^108^ semilocal functional with the Tkatchenko–Scheffler (TS)^109^ pairwise dispersion method. Pairwise methods add the dispersion contribution to the DFT total energy by summing over the interactions between pairs of atoms. In the TS method, the parameters of the correction are derived from the DFT charge density in a first-principles manner. Next, the structures are re-relaxed and re-ranked using PBE with the many-body dispersion (MBD) method.^110,111^ The MBD method considers the non-additive many-body contributions to the dispersion energy, as well as the effect of dielectric screening on the atomic polarizabilities. Finally, only the structures within the polymorph range are re-ranked by single-point energy evaluation using the more accurate but computationally expensive PBE-based hybrid functional (PBE0),^112^ which contains a fraction of exact (Fock) exchange in addition to the semilocal exchange and correlation. PBE0+MBD has been shown to provide accurate ranking of molecular crystal polymorphs.^79,113−115^ Here, we consider the energy range for viable polymorphs as 4 kJ/mol, based on the observation that nonconformational polymorphs are typically within 4 kJ/mol of each other.^65^ Larger energy differences, of up to10 kJ/mol, have been observed in some cases, in particular for conformational polymorphs;^65,116^ however, because tetracene is a very rigid molecule, we consider larger energy differences unlikely.

For the sake of simplicity and speed, Simple uses a variety of approximations. In particular, delocalization of the singlet state beyond two molecules, which can occur in molecular crystals,^25,27,28,34,35^ is neglected. Therefore, the prospective SF performance of all structures within the polymorph energy range of 4 kJ/mol is further assessed using many-body perturbation theory (MBPT), which describes excited-state properties in the solid state with periodic boundary conditions. Within MBPT, band structures are computed within the *GW* approximation, where *G* is the one-particle Green’s function and *W* is the screened Coulomb interaction.^117−119^ The *GW* approximation accounts for the renormalization of the electron energies due to the polarization response to the addition or removal of an electron.^120^ The optical properties, including singlet and triplet excitation energies, optical absorption spectra, and exciton wave functions, are subsequently calculated by solving the Bethe–Salpeter equation (BSE), using the *GW* quasiparticle energies as input. The BSE accounts for the electron–hole interaction and resulting exciton binding energy.^119,121,122^

#### GA Fitness Functions

To perform traditional CSP, GAtor uses an energy-based fitness function, in which structures with lower energies are assigned a higher fitness value. To evaluate the energy-based fitness function, the energy of the *i*th crystal relative to the GA pool, ϵ_*i*_, is computed using
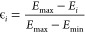
1where *E*_max_ and *E*_min_ are the maximal and minimal total energy values in the population, respectively, and *E*_*i*_ is the energy of structure *i*. The energy-based fitness, *f*_*i*_^energy^,^102^ is then computed as
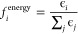
2

To steer the GA to explore undersampled low-energy regions of the potential energy surface, evolutionary niching has been implemented in GAtor by using a cluster-based fitness function. AP is used to cluster the population on the basis of structural similarity with respect to an RSF descriptor. The fitness of each cluster is divided by its number of members, such that oversampled basins are penalized. The population is re-clustered, and fitness is re-evaluated after the addition of each new structure to the pool. The cluster-based fitness function, *f*_*i*_^niching^,^103^ is given by
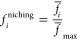
3with
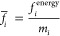
4where *m*_*i*_ is the niche count, i.e., the number of members in the cluster, to which the *i*th structure belongs.

Here, we have implemented a new fitness function to perform inverse design of the crystal packing to enhance the SF performance. To compute the SF-based fitness, the relative performance for the *i*th crystal, σ_*i*_, is defined as
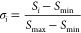
5where *S*_min_ and *S*_max_ are the minimum and maximum values, respectively, in the pool of the logarithm of the SF rate computed using Simple, as shown in Figure 2c. To simultaneously maximize the SF performance and minimize the lattice energy, we define the combined fitness as
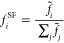
6with
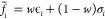
7where *w* is a weight factor that controls the relative importance of lattice energy and SF rate and ϵ_*i*_ is the relative energy, as defined in eq 1. Here, we use a *w* of 0.5.

### Computational Details

#### Genarris

An initial population of structures for GAtor was generated using the Robust Workflow implemented within Genarris,^73^ as shown in Figure 2a. The initial “raw” pool contained approximately 10,000 random structures with two molecules per unit cell (*Z* = 2). To prevent the generation of structures with unphysically close intermolecular contact distances, Genarris uses a specific radius proportion (*s*_r_). If atoms A and B with van der Waals radii *r*_A_ and *r*_B_, respectively, belong to two distinct molecules and are closer to each other than *s*_r_(*r*_A_ + *r*_B_), then the structure is rejected. For structure generation, we used an *s*_r_ of 0.85. Genarris predicted the unit cell volume to be 606 Å^3^, which differs from the experimental values of 580 Å^3^ for T1 and 573 Å^3^ for T2 by only a few percent. Next, the RSF descriptor was computed for all of the structures, and diversity-based selection was performed by clustering the structures using AP and selecting 1000 structures from the cluster centers (exemplars). Then, single-point energy evaluations were performed for the remaining structures using the PBE^108^ functional with the TS^109^ pairwise dispersion correction with the*lower-level* settings described in Section 2.2.4. AP clustering was performed again into 100 clusters, and the lowest-energy structure from each cluster was selected. Finally, the geometry of the remaining structures was optimized using PBE+TS with *lower-level* settings. Duplicate structures were removed from the pool using the Pymatgen Structure Matcher tool^123^ with the default tolerances, leaving 50 structures in the pool. Statistical analysis of the population of structures throughout the workflow of Genarris is provided in the Supporting Information.

#### GAtor

All GAtor runs were seeded using the same set of initial structures generated by Genarris. Six GAtor runs were conducted, as shown in Figure 2b, using the energy-based fitness function, the niching fitness function, and the SF-based fitness function. For each fitness function, two runs were performed using crossover to mutation probabilities of 25% and 75% and tournament selection with a tournament size of 10.^102^ “Standard mutation” and “standard crossover” schemes were used for mutation and crossover. The intermolecular closeness checks used an *s*_r_ of 0.8. Local optimizations and energy evaluations within GAtor were performed using PBE+TS with *lower-level* settings. Each run generated at least 60 structures. Energy convergence plots are provided in the Supporting Information. All of the runs together generated ∼400 structures, approximately half of which were found to be duplicates. The 100 lowest-energy structures were reoptimized and re-ranked using PBE+TS and PBE+MBD^110^ with the*higher-level* settings, described in Section 2.2.4. The crystal structures within the polymorph energy range were re-ranked again with PBE0+MBD and *higher-level* settings by single-point energy evaluations using the PBE+MBD geometries. The version of GAtor used for this work (GAtor 1.2) is freely available for download from https://www.noamarom.com/software/download/.

#### Simple

To assess SF rates, we used the Simple^61^ program for dimers extracted from the generated crystal structures of tetracene. All dimers with an intermolecular center of mass distance of <10 Å were considered. In Simple, the HOMO and LUMO are expanded using a natural atomic orbital (NAO) basis for computation. If hA, lA, hB, and lB denote the HOMO and LUMO of molecules A and B, respectively, then the first singlet state is represented by ^1^(hA → lA) and ^1^(hB → lB). The charge transfer states, where an electron is transferred, are given by ^1^(hB → lA) and ^1^(hA → lB), and the final biexciton is represented by ^3^(hA → lA) and ^3^(hB → lB), which couples into an overall singlet state. Natural atomic orbitals were computed using the natural bond orbital (NBO) analysis version 3.1 code within the Gaussian 16 package.^124^ The reorganization energy for computing SF rates was set to 0.3 eV.^62^ A Python script was used to create input files for Simple from NBO analysis and automate the calculation for all dimer geometries within a crystal. The logarithm of the highest SF rate among all of the dimers (*s*_*i*_) was used as a measure of the SF performance of a given crystal.

#### DFT

Genarris and GAtor interface with the FHI-aims^125^ code for geometry optimization and energy evaluation of crystal structures. The *lower-level* settings used within GAtor and Genarris correspond to the light numerical settings and tier 1 basis sets of FHI-aims. The *higher-level* settings used for re-ranking correspond to the tight numerical settings and tier 2 basis sets of FHI-aims. All calculations (except the interaction chain analysis provided in the Supporting Information) used a k-point mesh of 3 × 3 × 3.

#### *GW*+BSE

*GW*+BSE, as implemented in the BerkeleyGW code,^126^ was used here to evaluate the excited-state properties of putative tetracene polymorphs. To obtain the input wave functions for *GW*+BSE calculations, mean field DFT calculations using the PBE functional were performed with the Quantum ESPRESSO code.^127^ A coarse k-grid of 4 × 4 × 2 was used in the mean field calculations. We used Troullier–Martins norm-conserving pseudopotentials.^128^ The kinetic energy cutoff was set to 50 Ry. The RPA dielectric matrix and the electron self-energy within the *GW* approximation used the coarse grid wave functions as input. 548 unoccupied bands were included in the *GW* calculation. The BSE was solved within the Tamm–Dancoff approximation (TDA). Forty valence bands and 40 conduction bands were included in the BSE calculation. Taking the full dielectric matrix as input to screen the attraction between the electron (e) and hole (h), we constructed the e–h interaction kernel on the coarse k-point grid. To construct the Bethe–Salpeter Hamiltonian, the *GW* quasiparticle energies and e–h interaction kernel calculated with coarse k-point settings were interpolated onto the fine k-point grid of 8 × 8 × 4. The subsequent diagonalization yielded the excitation energies and wave functions. The exciton wave functions of the tetracene polymorphs were converged using supercells of 8 × 8 × 4, based on the criterion proposed in ref (34). Then, the degree of SF charge transfer character (%CT) of the singlet exciton was calculated by double-Bader analysis (DBA).^28,34^ DBA is an extension of the Bader charge partitioning scheme to exciton wave functions with two spatial variables. %CT is calculated by performing nested sums over the electron distributions obtained for different positions of the hole within a molecule. Absorption spectra were calculated for light polarized along the three crystal axes and averaged.

## Results and Discussion

### GA Performance

**Figure 3:**
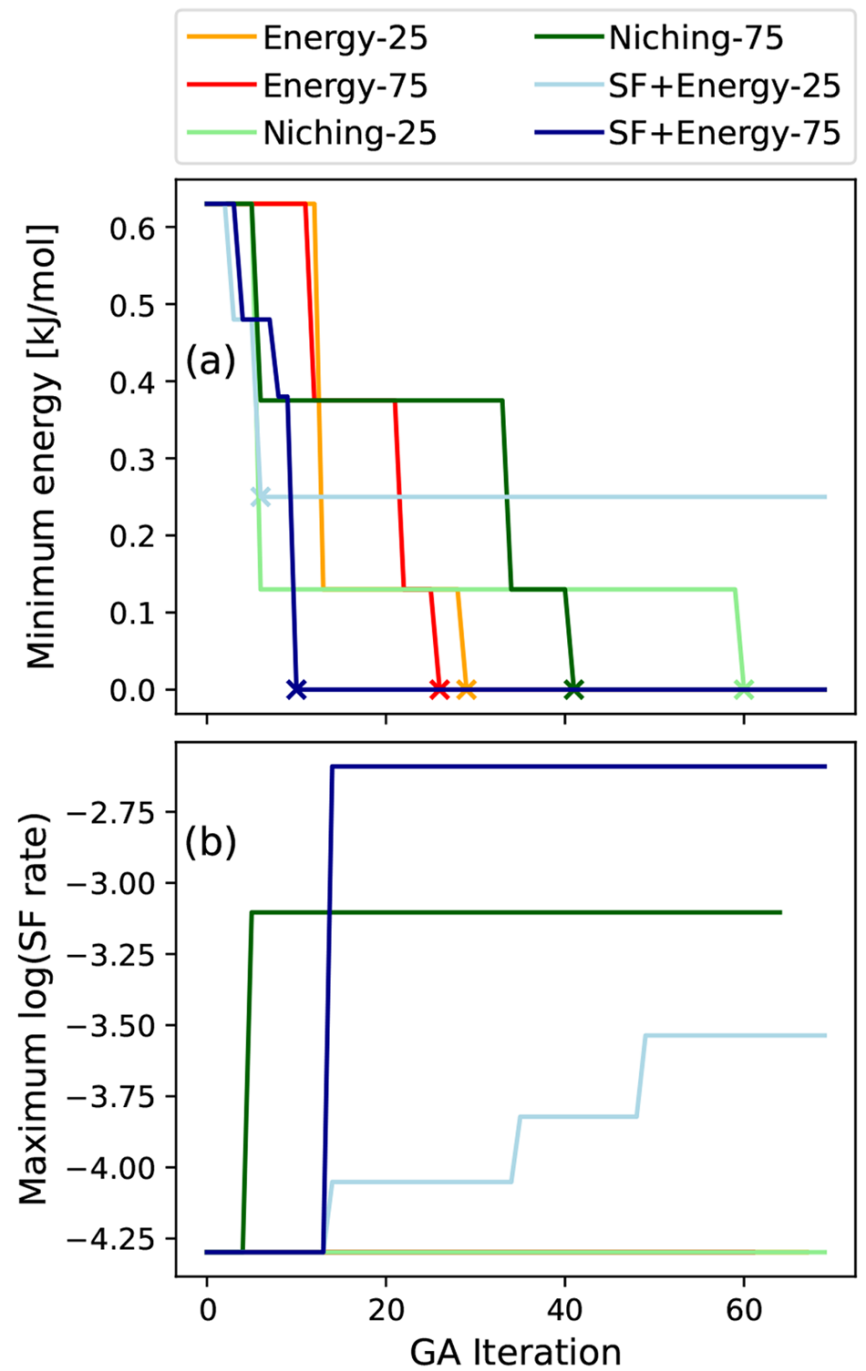
(a) The minimum energy and (b) the maximum SF rate in the pool after each GAtor iteration for different fitness functions and crossover to mutation probabilities. The energy-based fitness does not improve the SF rate of the pool with GA iterations. The cross marks the (distorted) T1 structure.

Figure 3a shows the minimum energy of the GA population as a function of GA iteration, where each addition of a new structure to the population is considered as an iteration.^102^ All GA runs converge to the common form of tetracene, T1, which is the lowest-energy structure. We note that the GA run using the SF+energy fitness function with a 75% crossover probability generates the T1 structure with a slightly distorted geometry, which is ∼0.25 kJ/mol higher in energy. The reason for this is that geometry relaxations are performed within GAtor with computationally efficient and less stringent *lower-level* settings. This structure is identified as a duplicate of the relaxed T1 structure by GAtor’s duplicate check and subsequently relaxes to the T1 structure in postprocessing with PBE+MBD and *higher-level* settings (we note that once the distorted T1 structure is generated GAtor’s duplicate checks prevent the addition of new T1 forms to the pool because of the presence of this structure). The GA runs using the SF+energy fitness function are the quickest to generate the T1 structure, within 6 and 10 iterations for crossover probabilities of 25% and 75%, respectively. The GA runs using the energy-based fitness functions find the T1 structure within 29 and 26 iterations for crossover probabilities of 25% and 75%, respectively. The runs using evolutionary niching are the slowest to generate the T1 structure within 60 and 41 iterations for crossover probabilities of 25% and 75%, respectively. The T2 structure is generated quickly by all GA runs. All runs generate it within five iterations, except the run using evolutionary niching with a crossover probability of 75%, which finds T2 within eight iterations.

**Figure 4:**
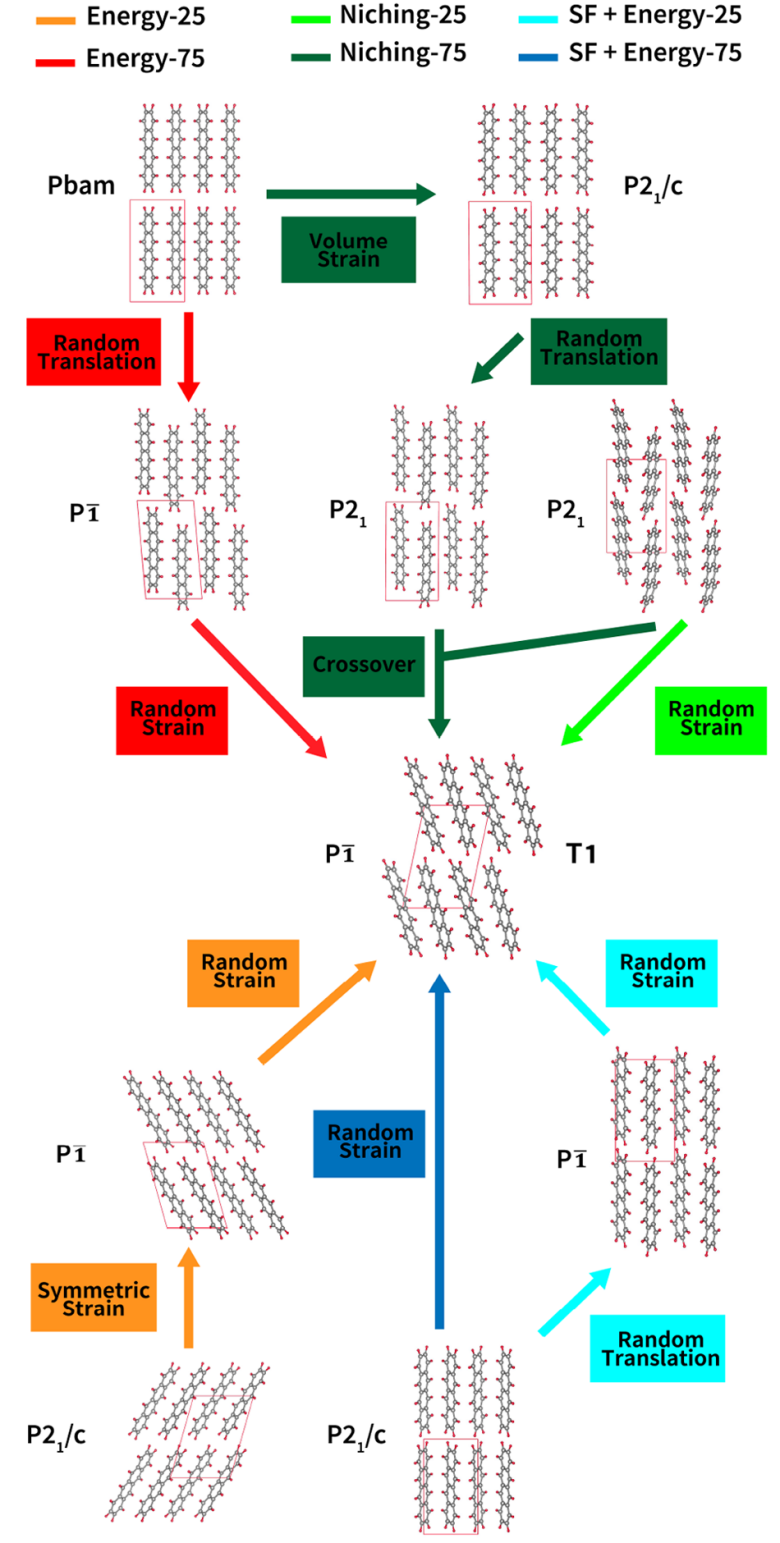
Evolutionary routes that produced the T1 experimental structure of tetracene in GA runs using different fitness functions and crossover probabilities. All routes start from initial pool structures. The packing motifs and space groups of all structures are also shown. The *a*-, *b*-, and *c*-axes are colored red, green, and blue, respectively.

**Figure 5:**
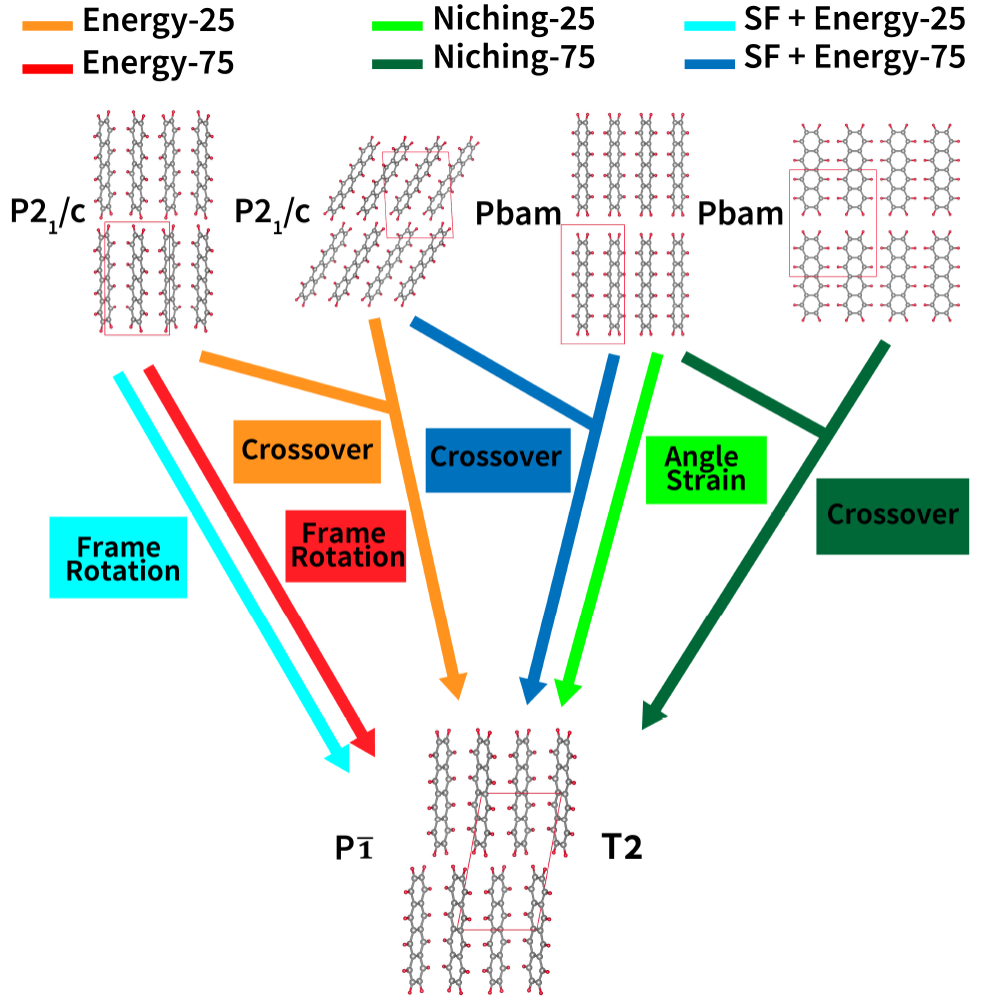
Evolutionary routes that produced the T2 experimental structure of tetracene in GA runs using different fitness functions and crossover probabilities. All routes start from initial pool structures. The packing motifs and space groups of all structures are also shown. The *a*-, *b*-, and *c*-axes are colored red, green, and blue, respectively.

Each GA run follows its own unique path to reach the experimental structures. Figures 4 and 5 show the different evolutionary routes traversed by GAtor runs using different fitness functions and crossover to mutation probabilities. The T1 structure is generally reached by complex routes, typically consisting of more than one step. Most routes involve only mutation operations, in particular the strain mutation. In contrast, the routes for generating T2 involve only one step of either mutation or crossover from the initial pool. This explains why the T2 structure is generated fast by all GA runs. For T2, both types of breeding operators, crossover and mutations, are equally beneficial. For both the T1 and T2 structures, the initial pool ancestors have higher space group symmetries that are broken by the GA breeding operators.

Figure 3b shows the maximum SF rate in the population as a function of GA iteration. As explained in Methods, the SF rate of a given crystal structure is taken as the logarithm of the highest SF rate computed by Simple out of all of the dimers extracted from that structure. For the GA runs using the energy-based fitness function, the maximum SF rate does not improve relative to the initial pool. In contrast, both GA runs using the SF+energy-based fitness function generate packing motifs comprising dimers that yield higher SF rates. This is because higher fitness is assigned to structures that have desirable dimers. Consequently, these structures are selected more often for breeding and pass their structural genes to offspring via the mutation and crossover operations. This improves the average SF rate of the population resulting in more optimal structures for SF. The GA runs using evolutionary niching produce mixed results. The run using a crossover probability of 25% does not generate structures with SF rates that are higher than those in the initial population, whereas the run using a crossover probability of 75% does. While the niching fitness function is not specifically tailored to search for structures with higher SF rates, it is designed to perform more exploration of undersampled regions of the PES. Thus, it may fortuitously stumble upon structures with higher SF rates.

**Figure 6:**
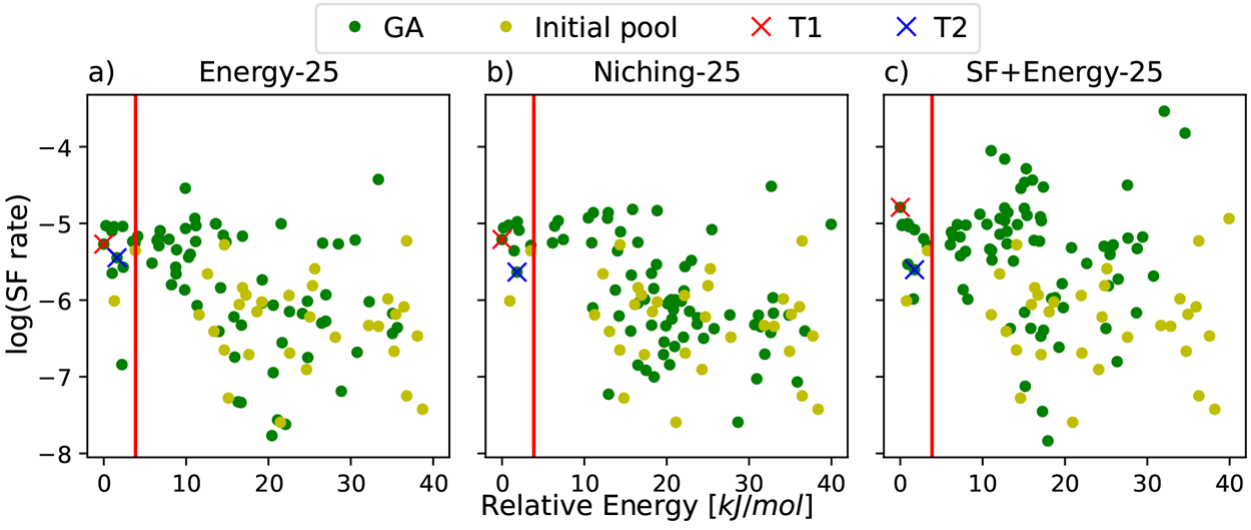
SF rates as a function of relative energy for the structures generated by GAtor runs with a crossover probability of 25% using (a) the energy-based fitness function, (b) evolutionary niching, and (c) the SF+energy-based fitness function. The red line denotes the polymorph range of 4 kJ/mol. The T1 and T2 polymorphs of tetracene are indicated by red and blue crosses, respectively. The relative energies presented here are as computed during the GAtor runs using PBE+TS with *lower-level* settings. Slight differences in the relaxed geometries at this level of theory produce somewhat different SF rates for the T1 and T2 structures generated in different GA runs.

Figure 6 shows the SF rate computed by Simple as a function of the relative energy of the crystal structures generated by the GAtor runs using a crossover probability of 25% with different fitness functions. Plots for the runs using a crossover probability of 75% are provided in the Supporting Information. The T1 and T2 forms are indicated by red and blue crosses, respectively. We note that the relative energies presented here are as computed during the GAtor runs using PBE+TS with *lower-level* settings. SF rates of dimers computed using Simple are known to be highly sensitive to the geometry.^62^ Therefore, slight differences in the relaxed geometries at this level of theory produce somewhat different energies and SF rates for the T1 and T2 structures generated in different GA runs. This emphasizes the need to re-relax the GA-generated structures with *higher-level* numerical settings during postprocessing to obtain more accurate and reliable geometries for final energy ranking, as discussed below (see additional analysis of the dependence of the Simple rate on the geometry in the Supporting Information). Compared to the runs using the energy-based fitness function and evolutionary niching, the run using the SF+energy fitness function generates a significantly larger number of structures with a SF rate higher than that of the structures found in the initial population. However, most of the structures with particularly high SF rates are outside of the polymorph range of 4 kJ/mol, indicated by the red line. This is likely because the packing motifs that comprise dimers with a high SF rate are not energetically favorable. A similar behavior is evident in the runs using 75% crossover probability, as shown in the Supporting Information.

**Figure 7:**
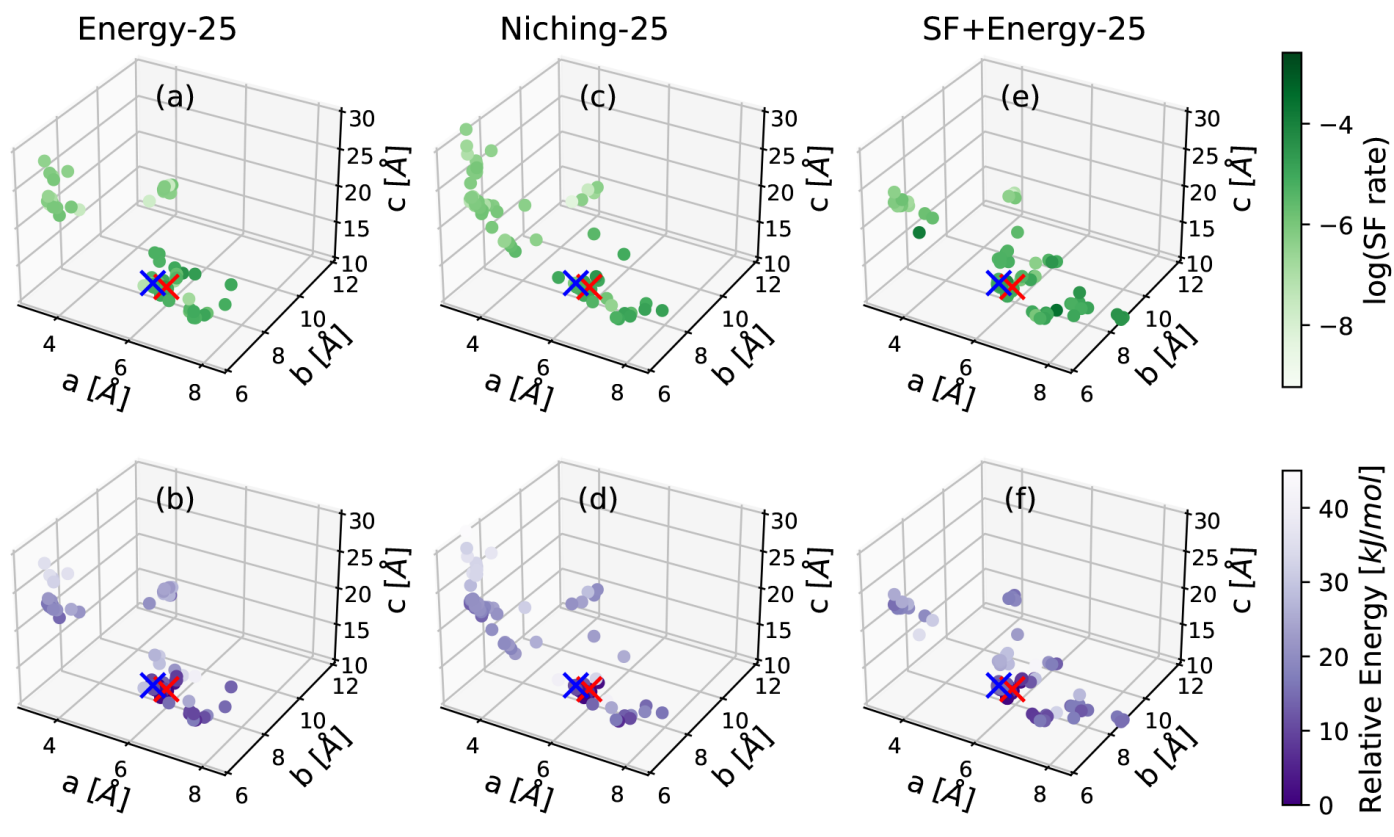
Lattice parameter distributions of the structures generated by GAtor runs with different fitness functions and 25% crossover to mutation probability. Panels a and b present the SF rate and relative energy, respectively, as a function of lattice parameters for the GA run using the energy-based fitness function. Panels c and d present the SF rate and relative energy, respectively, as a function of lattice parameters for the GA run using evolutionary niching. Panels e and f present the SF rate and relative energy, respectively, as a function of lattice parameters for the GA run using the SF+energy-based fitness function. The T1 and T2 polymorphs of tetracene are marked by red and blue crosses, respectively.

Figure 7 shows the lattice parameter distributions of the structures generated by the GA runs using different fitness functions with a 25% crossover probability. The lattice parameter distributions for the runs with a 75% crossover probability are shown in the Supporting Information. The T1 and T2 structures are marked by red and blue crosses, respectively. The structures are colored based on the basis of the logarithm of their Simple SF rates and their PBE+TS energy relative to the T1 structure in the top and bottom rows, respectively. The T1 and T2 structures are located in the same basin of the lattice parameter space. Overall, the lattice parameter space has a single low-energy basin, in which all of the potential polymorphs of tetracene reside. In contrast, there are multiple high-SF rate regions outside of the low-energy cluster. As shown in panels a and b, the GA run using the energy-based fitness function mainly explores the main low-energy basin. The GA runs using evolutionary niching and the SF-based fitness function both explore more outside of the main low-energy basin. However, the regions they explore differ. The niching run heavily explores a region on the left side of the lattice parameter plot with small *a* parameters, which turns out to be unproductive because it does not contain structures with a particularly low energy or high SF rate. This explains why the niching run is the slowest to generate the experimental structures. In the case of tetracene, the main low-energy basin also coincides with relatively high SF rates. This is why the run using the energy+SF-based fitness function succeeds in finding both experimentally known structures relatively fast. This run also explores a region on the bottom right of the lattice parameter plot, characterized by several clusters with relatively high SF rates. The exploration of these regions of the configuration space explains why the GA run using the SF+energy-based fitness function succeeds in generating more structures with relatively high SF rates. As shown in the Supporting Information, the runs with a crossover probability of 75% also show a similar trend. We note that the structure of the PES, namely, the number of low-energy basins and whether they coincide with high-SF rate regions, is dependent on the material. Therefore, for materials other than tetracene, the different fitness functions and GA operations may perform differently in terms of the speed of finding the known structure(s) and other putative polymorphs with enhanced SF performance.

**Figure 8:**
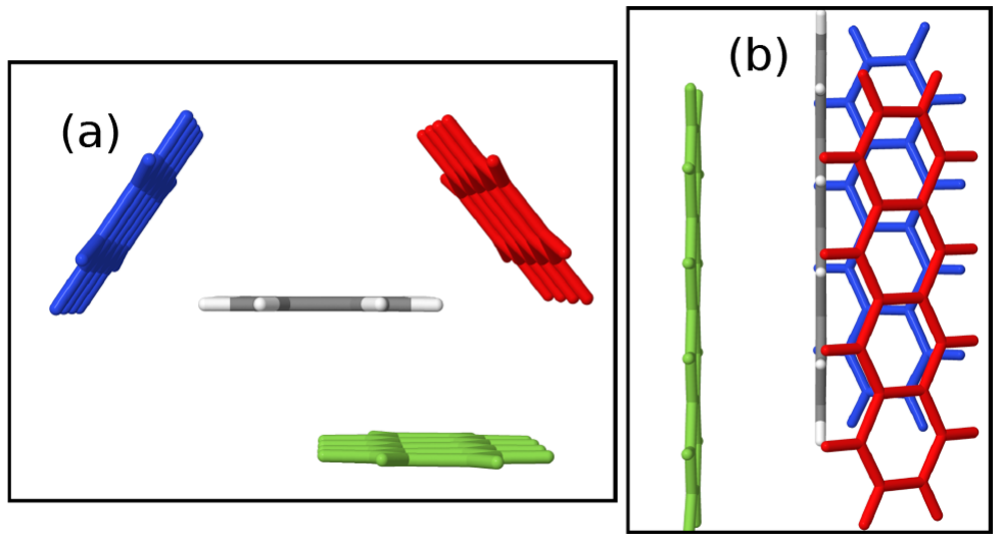
Comparison of the highest SF dimers of different crystal structures viewed along directions of: (a) the in-plane long axis and (b) the in-plane short axis of the tetracene molecule. T1 is colored red. T2 is colored blue. The structure with the overall highest-rate SF dimer in the GA pool is colored lime green.

Figure 8 shows a comparison between dimers extracted from the structure with the highest overall Simple SF rate and from the T2 and T1 forms. The best overall dimer, colored lime green in Figure 8, has a slip-stacked structure with the molecular planes facing each other. This dimer belongs to a crystal structure that has a π-stacked packing motif with a slip in the direction of the in-plane short axis. This packing arrangement is not energetically favorable for tetracene, with the energy of this structure being over 20 kJ/mol above the global minimum energy, as shown in Figure 6c. Therefore, it is unlikely to be experimentally synthesizable. In the dimers extracted from the T1 and T2 structures, the molecules are positioned at an angle, with no cofacial interactions. This is a result of the herringbone packing of these structures.

Previously, the best dimers of tetracene have been identified and ranked using a grid search approach with Simple.^62^ Therein, the dimers were isolated, not a part of a crystal structure, and their relative stability was not considered. The dimer with the highest SF rate generated by GAtor resembles the fifth best tetracene dimer found in ref (62). The best dimer extracted from the T1 and T2 structures ranks as the 10th best dimer in ref (62). In most of the best SF dimers found in the GA population, the molecules have their long axes parallel to each other. In contrast, in the best isolated dimers found in ref (62), the molecules are rotated along the out-of-plane axis, such that their long axes are non-parallel. Such configurations are uncommon among the low-energy crystal structures of tetracene, presumeably because they are less stable.

On the basis of the results shown in Figures 3–8, the SF+energy fitness function successfully biases the GA toward generating dimers with higher SF rates that are also energetically stable within a crystal structure. Structure–property relations can be derived by examining the structures predicted to have high SF rates. Although some of the packing motifs with dimer configurations that produce high SF rates are not energetically favorable for tetracene, valuable insight is still gleaned as to what packing arrangements should be sought. It may be possible to achieve such packing arrangements by chemical modification of the tetracene backbone. For example, it has been shown that adding side groups can help stabilize different packing motifs.^45,129−131^

### Putative Polymorphs

**Figure 9:**
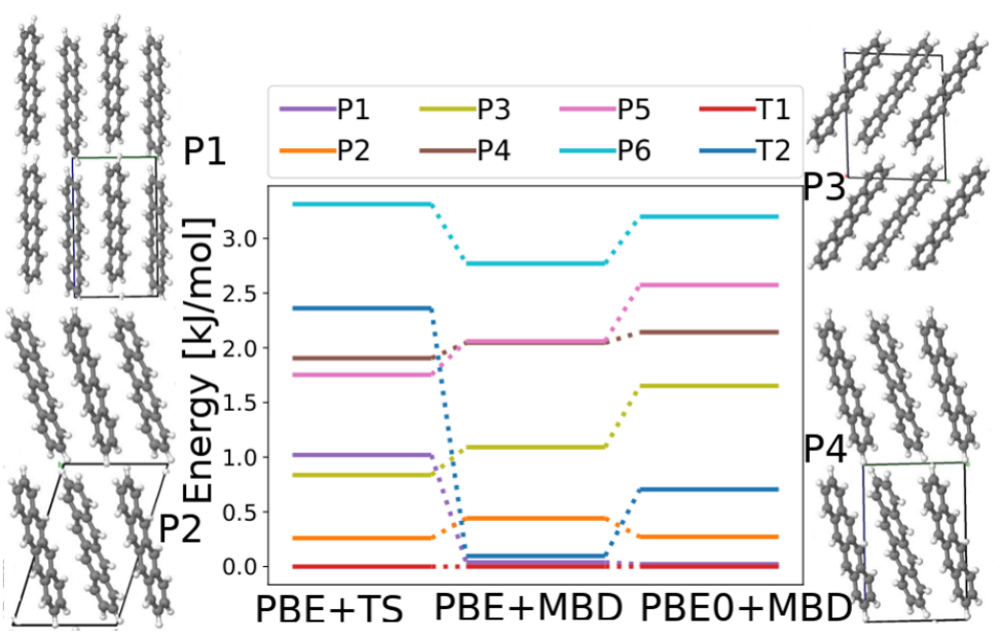
Re-ranking of tetracene structures generated by GAtor using increasingly accurate DFT functionals and dispersion methods. Relative energies are referenced to the lowest-energy structure with each method. The experimental structures and some low-energy putative structures are also shown.

After combining the structures generated by all GAtor runs, re-relaxing them with higher-level numerical settings, and performing duplicate checks, about 160 unique structures remain. These structures undergo postprocessing, as described in Methods. Figure 9 shows the energy ranking of the structures in the polymorph range of 4 kJ/mol using increasingly accurate exchange-correlation functionals and dispersion methods. The experimentally stable form, T1, is consistently ranked as the lowest in energy by all three methods. The ranking of the T2 form changes significantly upon switching from the TS pairwise dispersion method to the MBD method. With PBE+TS, the T2 structure is found ∼2.5 kJ/mol above the T1 structure, whereas PBE+MBD ranks T1, T2, and P1 as nearly degenerate within an energy range of 0.2 kJ/mol. Switching from the semilocal PBE functional to the PBE0 hybrid functional destabilizes the T2 structure and shifts it to ∼0.75 kJ/mol above the T1 form. To further investigate the differences between DFT functionals and dispersion methods, we performed interaction chain analysis, as described in ref (104), for the T1 and T2 structures. A full account is provided in the Supporting Information. We find that side-to-face and face-to-face interactions, which are present in both the T1 and T2 structures, are overstabilized by PBE+TS and understabilized by PBE+MBD, compared to PBE0+MBD. Edge-to-edge interactions, which are present only in the T2 structure (along the *c*-axis), are overstabilized by both PBE+TS and PBE+MBD, compared to PBE0+MBD. Whether a certain structure is understabilized or overstabilized compared to another depends on the overall balance of different interactions. As shown in the Supporting Information, for tetracene, stability is loosely correlated with density. Several putative structures are as dense but significantly less stable than the structures in the polymorph range.

Because of its computational efficiency, Simple is a practical choice for evaluating the SF performance of putative crystal structures within the GA. However, as explained above, the dimer model implemented in Simple does not consider the extended nature of excitons in molecular crystals, which may be delocalized over several molecules, as is the case for tetracene.^57^ For tetracene, the best dimer extracted from the T1 structure is predicted to yield a SF rate higher than that of the T2 structure (see Figure 6), contrary to experimental observations.^57^ We note that in the future Simple may be replaced by more advanced models that go beyond the dimer approximations and/or by machine learning models^36^ for the evaluation of the SF-based fitness function. The GW+BSE method can capture the many-body effects in a molecular crystal with periodic boundary conditions. We proceed to evaluate the prospective SF performance of structures by using GW+BSE to calculate the singlet and triplet excitation energies and the corresponding exciton wave functions. The GW+BSE calculations were performed only on structures in the polymorph range due to their high computational cost.

**Figure 10:**
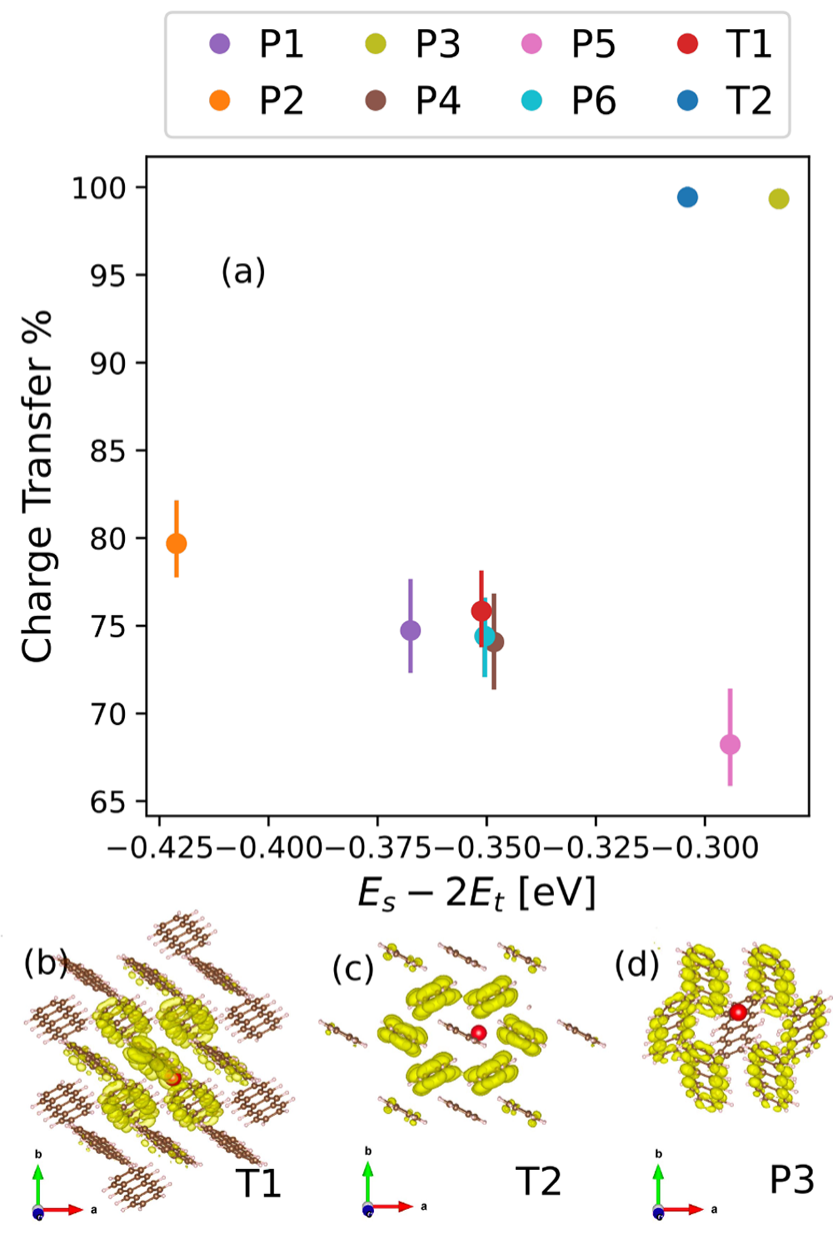
(a) Putative polymorphs of tetracene compared to the T1 and T2 structures based on a two-dimensional descriptor calculated with *GW*+BSE. The thermodynamic driving force for SF, *E*_S_ – 2*E*_T_, is displayed on the *x*-axis, and the singlet exciton charge transfer character, %CT, is displayed on the *y*-axis. The error bars represent the range of %CT values obtained using double-Bader analysis with the hole located at different sites. Exciton wave functions for (b) T1, (c) T2, and (d) P3 are also shown. The electron distribution is colored yellow with respect to the hole position colored in red.

In Figure 10, the structures in the polymorph range are compared to the two known forms of tetracene with respect to a two-dimensional descriptor for SF performance, evaluated using *GW*+BSE.^27,28,33,34^ The primary descriptor, plotted on the *x*-axis, is the thermodynamic driving force for SF, i.e., the energy difference between the singlet exciton energy and twice the triplet exciton energy, *E*_S_ – 2*E*_T_. We note that the singlet and triplet excitation energies obtained with *GW*+BSE are vertical values, neglecting geometry relaxation in the excited state. Vibrational effects and entropic effects are also not considered here. Because *GW*+BSE systematically underestimates the SF driving force,^27,28,33−35^ we restrict the discussion to a comparative assessment of the putative polymorphs. A high driving force indicates that a material is likely to undergo SF with a high rate. However, an overly high driving force increases the energy loss in the SF process and may be beneficial to other processes competing with SF. In the T1 structure of tetracene, SF is slightly endoergic, which leads to a relatively slow fission rate.^8,132,133^ Therefore, a driving force somewhat higher than that of the T1 structure of tetracene would be ideal.^5^

The singlet exciton wave functions of T1 and T2, computed using *GW*+BSE, are shown in Figure 10. Exciton wave functions have two spatial variables corresponding to the electron and hole probability distributions. Here, the electron probability distribution is visualized in yellow with respect to a fixed hole position, colored in red. Excitons in molecular crystals may be classified on the basis of the localization of the electron distribution with respect to the hole position.^6^ In a Frenkel exciton, the electron distribution is concentrated on the same molecule as the hole. In a charge transfer (CT) exciton, the electron is distributed on other molecules. Typically, excitons are not purely one or the other but have a degree of charge transfer character (%CT). The degree of CT character of the singlet exciton wave function is the secondary descriptor displayed on the *y*-axis in Figure 10. This descriptor is motivated by the growing body of experimental evidence for the involvement of a virtual charge transfer state in the SF process.^29,134−137^ A singlet exciton with a high degree of CT character, i.e., with the hole and the electron probability distributions centered on different molecules, is thought to be favorable for SF.^6,9,25,29,138,139^ For the singlet exciton of T1, there is some probability of finding the electron on the same molecule as the hole. In comparison, for T2 and P3 there is virtually no electron probability on the molecule with the hole.

**Figure 11:**
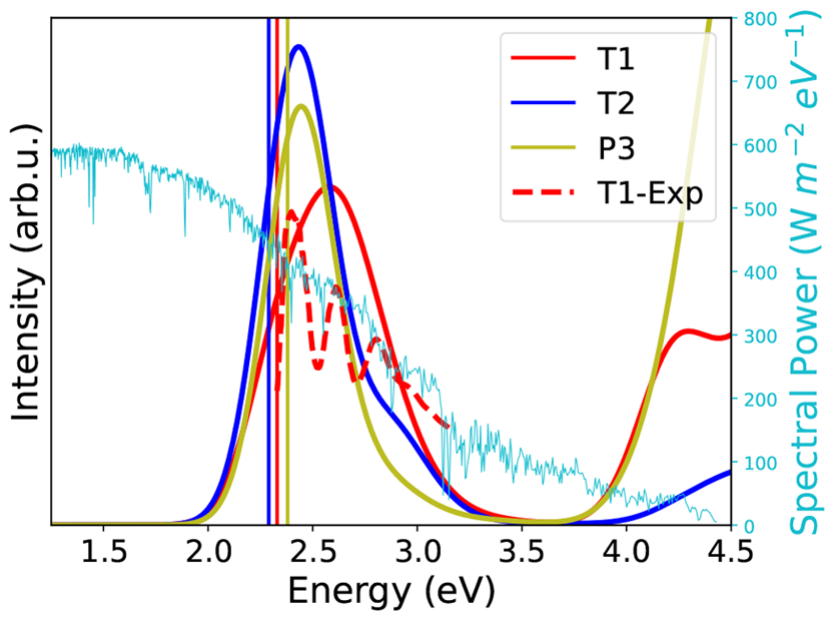
Absorption spectra of the T1, T2, and P3 structures computed using *GW*+BSE. The spectrum of T1 is compared with experimental data, adapted with permission from ref (140), Copyright 2008 John Wiley and Sons. The vertical lines denote the optical gap, which is equal to the lowest singlet excitation energy. The solar spectral energy distribution is also shown.

Crystal packing affects both the SF driving force and the character of the singlet exciton. The T2 structure has both a higher driving force and a singlet exciton with a higher degree of charge transfer character than the T1 structure. Hence, on the basis of the two-dimensional (2D) descriptor, the SF performance of the T2 structure is expected to be better than that of the T1 structure, which is consistent with experimental observations. Of the other structures in the polymorph range, P1 and P2 have a lower SF driving force than T1 and %CT values similar to that of T1. P4 and P6 have a driving force and a %CT similar to those of T1. P5 has a driving force higher than those of T1 and T2 but a %CT lower than that of T1. Only P3 has a higher driving force than both T1 and T2, as well as a high %CT, similar to that of T2. Thus, P3 is identified as the most promising polymorphic form of tetracene based on the 2D descriptor. In Figure 11, the absorption spectrum of the P3 structure, calculated with *GW*+BSE, is compared with the T1 and T2 structures. The optical gaps of all three structures are within a narrow range of 2.29–2.38 eV. However, the absorption characteristics near 2.5 eV are slightly different. T2 and P3 have a sharper absorption peak relative to T1. On this basis, the absorption behavior of the P3 structure is similar to that of the T2 structure and well-suited for SF-based solar cells. Moreover, the PBE0+MBD relative energy of P3 is only 1.5 kJ/mol above the T1 form, making it a potentially realizable structure.

## Conclusion

In summary, we have conducted inverse design of the crystal packing of tetracene to enhance its singlet fission performance. This was achieved by implementing a fitness function based on the SF rate and stability in the GAtor genetic algorithm package. For fast evaluation of SF rates within the GA, we used a dimer model implemented in the Simple code. We have demonstrated that the property-based genetic algorithm succeeds in generating structures predicted to have higher SF rates. Analysis of these structures reveals structure–property relations and provides insight into packing motifs associated with high SF rates. The structures found within the polymorph energy range of 4 kJ/mol above the common form of tetracene, which is the global minimum, were further assessed using many-body perturbation theory. We have identified a putative polymorph predicted to have a thermodynamic driving force for SF higher than those of both known forms of tetracene, and a singlet exciton with a high degree of charge transfer character. This structure is only 1.5 kJ/mol higher in energy than the common form of tetracene, well within the viable polymorph range. Therefore, it may be experimentally synthesizable.

Because of the versatility of the genetic algorithm, different fitness functions can be easily implemented. The SF-based fitness function may be improved in the future by using more advanced models that go beyond the dimer approximation and/or machine learning models.^36^ Furthermore, GA fitness functions may be tailored to search for any property or combination of properties of interest. The results of property-based GAs can help guide experimental synthesis efforts in promising directions. If metastable structures are found that are predicted to have desirable properties and be relatively close in energy to the global minimum, it may be possible to synthesize them. Specifically for molecular crystals, a variety of experimental techniques exist for growing metastable polymorphs,^89^ including changing the solvent and crystallization conditions (temperature, pressure, etc.),^90−92^ tailor-made additives,^93,94^ solution shearing,^95,96^ epitaxial templating,^97^ and nanoscale confinement.^98−101^ If packing motifs correlated with desirable properties are predicted to be too high in energy to be synthesizable for a parent compound, they may be achieved by chemical modifications, such as functionalization with side groups.^45,129−131,141^ We note, however, that CSP often produces more putative structures than are realized experimentally.^142^ This is mainly because crystallization conditions and kinetics are not considered in CSP, including the energy barriers for solid-state transformations.^143^ In conclusion, inverse design by property-based genetic algorithms is a highly useful strategy for discovery of materials with enhanced properties for various applications.

## Supporting Information Available

Statistical analysis of the population of structures throughout the Genarris workflow, additional analysis of GAtor runs, *GW* band structures and BSE absorption spectra of structures within the polymorph energy range, interaction chain analysis of the T1 and T2 structures, comparison of Simple rates before and after postprocessing (PDF)
Structures of the putative polymorphs found up to 10 kJ/mol above the global minimum in the form of FHI-aims geometry (ZIP)
