# Supplementary material for: Inverse Design of Tetracene Polymorphs with Enhanced Singlet Fission Performance by Property-Based Genetic Algorithm Optimization
Source: Chem Mater. 2023 Jan 21;35(3):1373–86. doi: 10.1021/acs.chemmater.2c03444 (PMC10042130; doi:10.1021/acs.chemmater.2c03444)
Supplement: Supplementary file 1 — cm2c03444_si_001.pdf [file cm2c03444_si_001.pdf]

Supplementary Informaiton: Inverse Design of Tetracene Polymorphs with Enhanced Singlet Fission Performance by Property-Based Genetic Algorithm Optimization

Rithwik Tom,† Siyu Gao,‡ Yi Yang,‡ Kaiji Zhao,‡ Imanuel Bier,‡ Eric A. Buchanan,¶ Alexandr Zaykov,§,‖ Zdeněk Havlas,§ Josef Michl,¶,§ and Noa Marom*,†,‡,⊥

†*Department of Physics, Carnegie Mellon University, Pittsburgh, PA 15213, USA*

‡*Department of Materials Science and Engineering, Carnegie Mellon University, Pittsburgh, PA 15213, USA*

¶ *Department of Chemistry, University of Colorado, Boulder, Colorado 80309, United States*

§ *Institute of Organic Chemistry and Biochemistry, Czech Academy of Sciences, 16610 Prague 6, Czech Republic*

‖ *Department of Physical Chemistry, University of Chemistry and Technology, 166 28 Prague 6, Czech Republic*

⊥*Department of Chemistry, Carnegie Mellon University, Pittsburgh, PA 15213, USA*

E-mail: nmarom.cmu.edu

Genarris

The Robust worflow of Genarris begins with generation of 10,000 random structures .[2] The two major principle components of atom-centered symmetry functions of structures in the pool shown in Fig. S2. A significant density of structures in the region near the experimental crystal that is maintained during different stages of the workflow. This indicates that Genarris has adequately sampled the configuration space near the experimental form with the given settings. Tetracene has D_2h point group symmetry which enables generation in multiple space groups. Space group distribution show sufficient sampling of $P\bar{1}$, the space group of T1 and T2 (Fig. S1). Unit cell volumes across different stages of the Genarris workflow are almost centered near the experimental values. These observations are indicative of a good quality Genarris run, with reasonable sampling around the experimental regions and within a small energy range of $25kJ/mol$. The lowest energy crystal structure from the pool, P3, is only 1 kJ/mol higher in energy relative to T1 using PBE+MBD. Finally, duplicate checks were performed to select 50 unique tetracene crystal structure. which constituted the initial pool for GAtor.

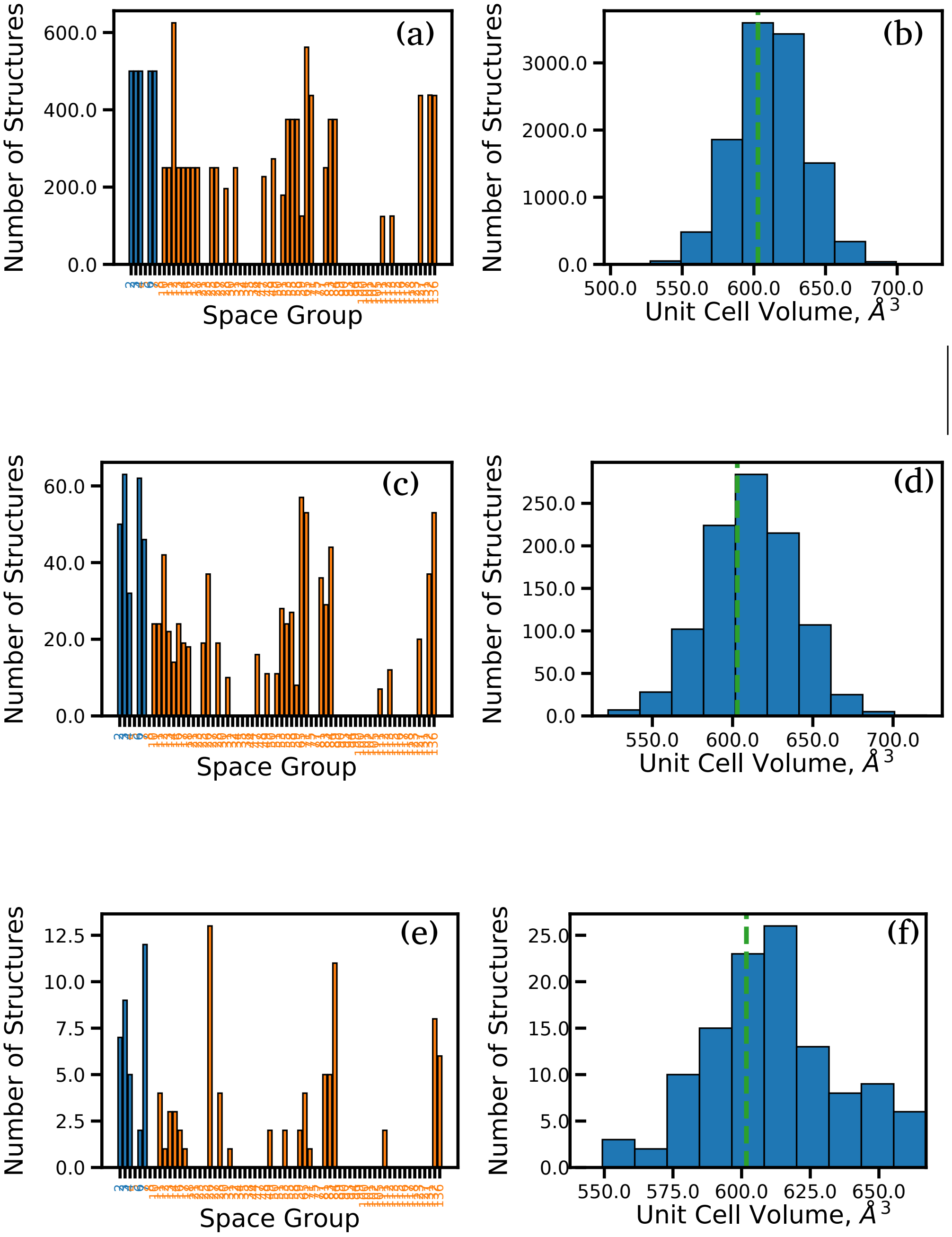

Figure S1: Space group distribution ((a), (c), (d)) and unit cell volumes ((b), (d), (f)) in different stages of the Genarris workflow. (a) and (b) corresponds to the raw pool, (c) and (d) corresponds to the first down-selection (AP1 pool), (e) and (f) corresponds to the last down-selection (AP2 Pool). Space groups with tetracene in special positions are marked in orange. The green line in the unit cell volume distribution correspond to the cell volume of the T1.

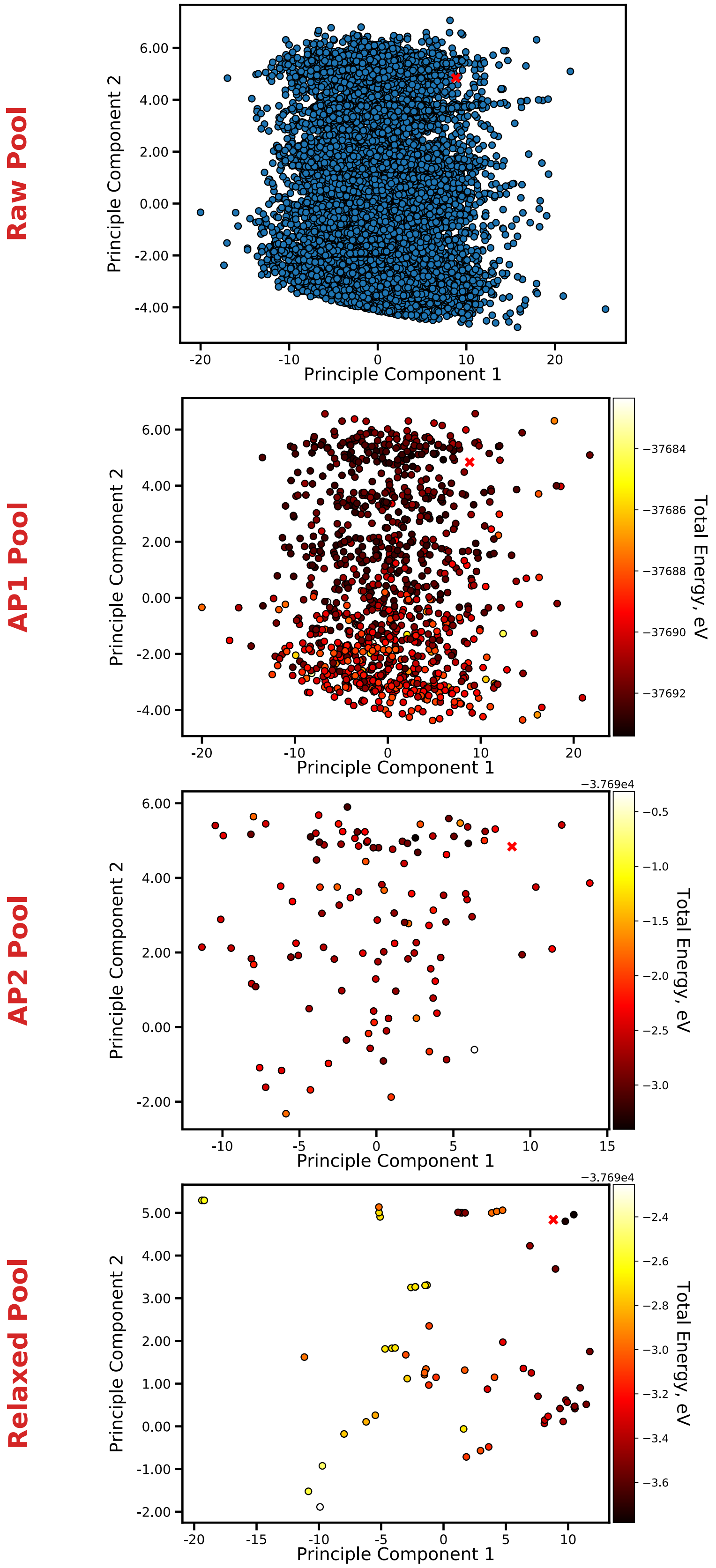

Figure S2: Principal component analysis (PCA) during different stages of Genarris .[1] The red cross denotes T1.

GAtor

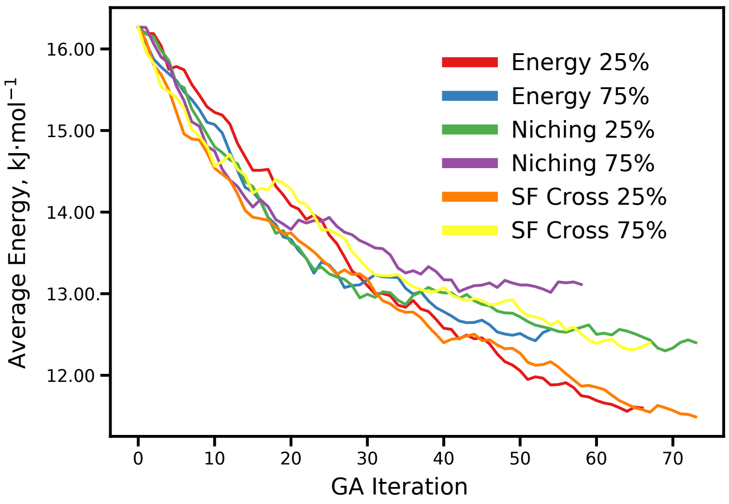

Figure S3: Average energy of GA runs with new structures added to the pool for different runs.

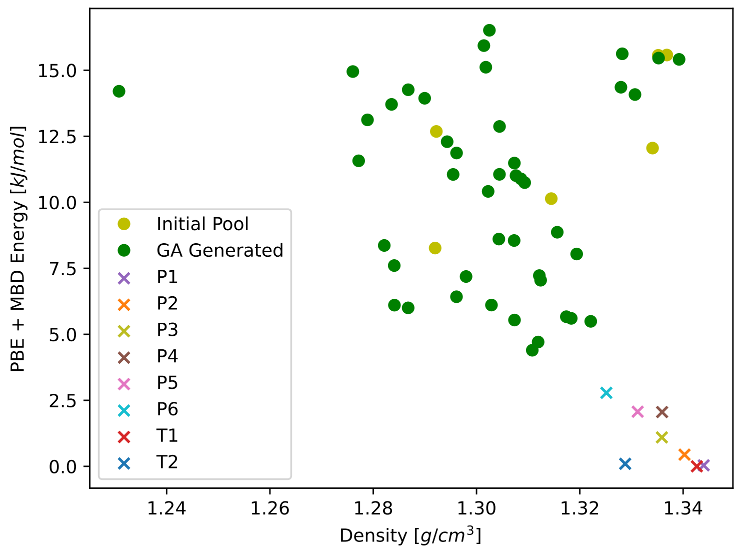

Figure S4: Plot of density vs the energy of low-energy tetracene crystals combining all GAtor runs. The initial pool refers to structures generated by Genarris whereas the rest of them are generated by GAtor.

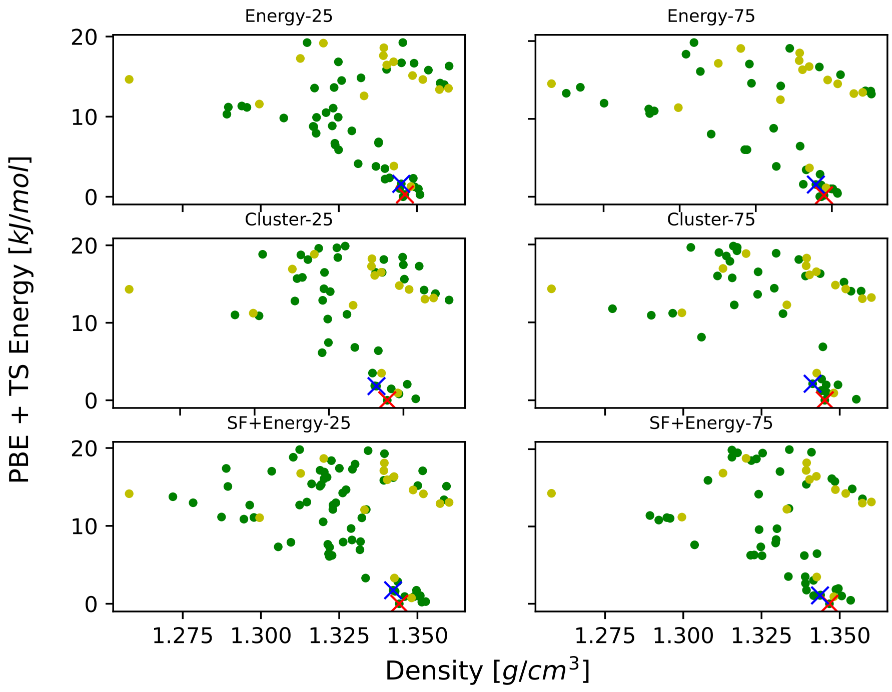

Figure S5: Plot of density vs the energy of low-energy tetracene crystals combining all GAtor runs. The initial pool are structures generated by Genarris whereas the rest of them are generated by GAtor. The two polymorphs of tetracene are marked in red (T1) and blue (T2).

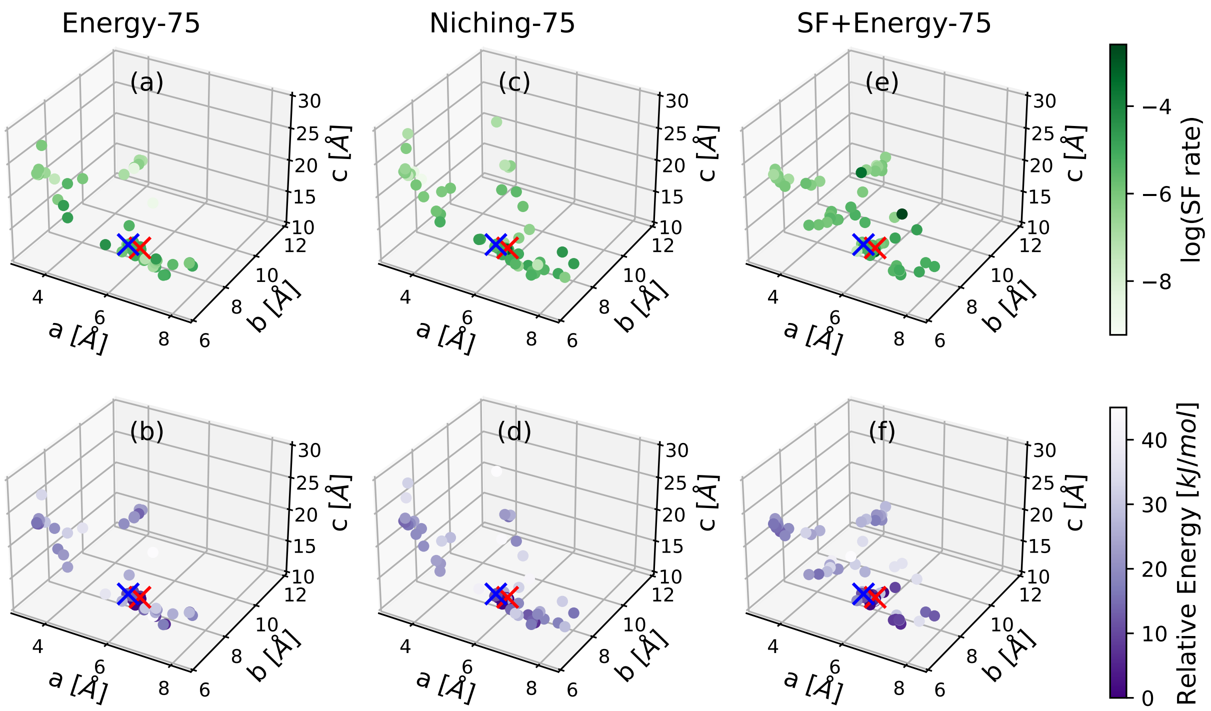

Figure S6: Lattice parameter distributions of the structures generated by GAtor runs with different fitness functions and 75% crossover to mutation probabilities. Panels (a) and (b) present the SF rate and relative energy, respectively, as a function of lattice parameters for the GA run using the energy-based fitness function; Panels (c) and (d) present the SF rate and relative energy, respectively, as a function of lattice parameters for the GA run using evolutionary niching; Panels (e) and (f) present the SF rate and relative energy, respectively, as a function of lattice parameters for the GA run using the SF+energy-based fitness function. The T1 and T2 polymorphs of tetracene are marked by red and blue crosses, respectively.

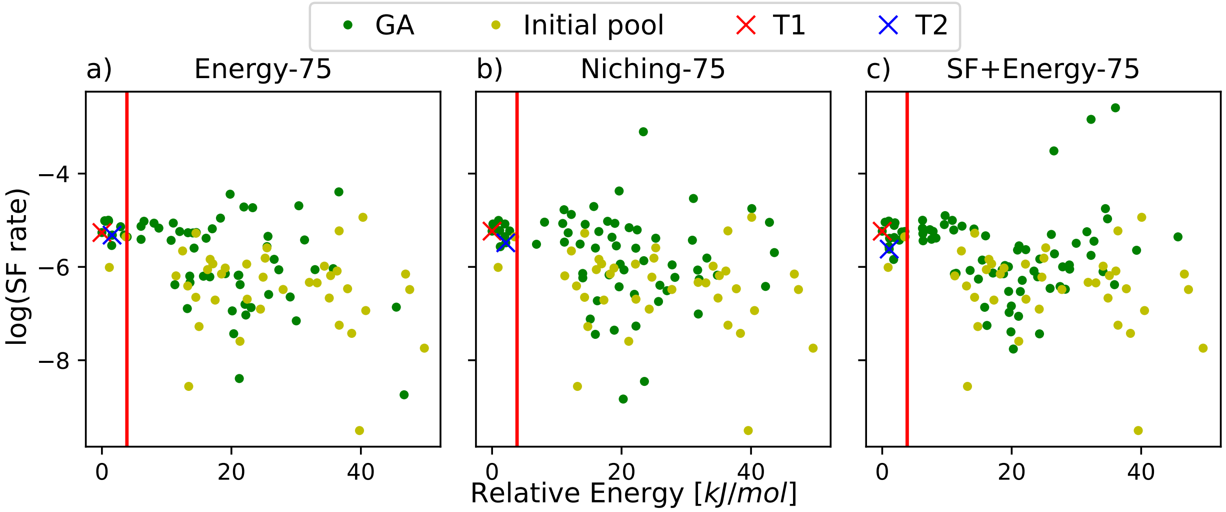

Figure S7: SF rate as a function of relative energy for the structures generated by GAtor runs with crossover probability of 75% using (a) the energy based fitness function, (b) evolutionary niching, and (c) the SF+energy based fitness function. The two polymorphs of tetracene are marked in red (T1) and blue (T2).

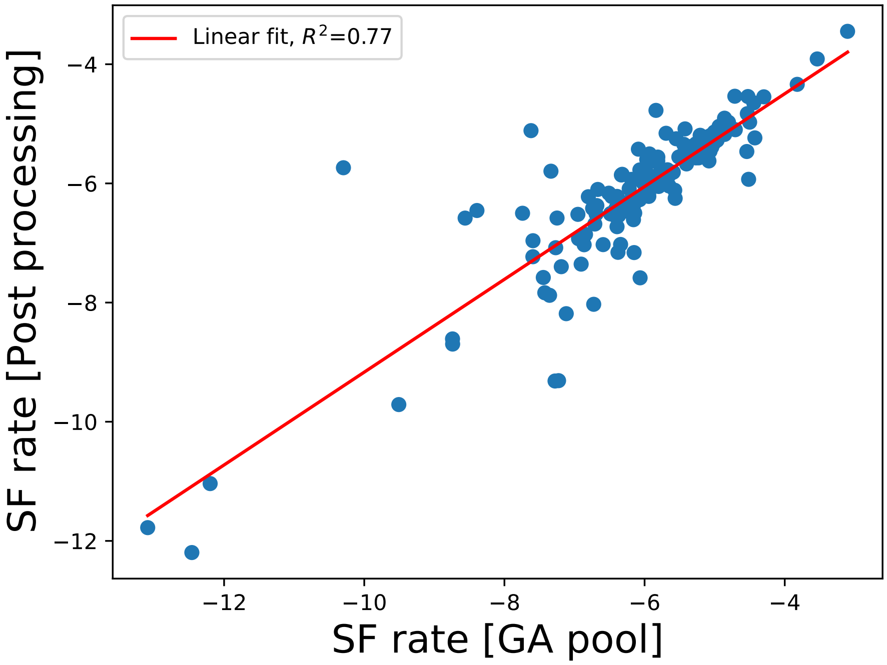

Figure S8: SF rate of the refined pool using PBE+MBD and *higher-level* settings plotted against SF rate of the pool generated by GAtor using PBE+TS using *lower-level* settings. The plot is in log scale. We note that the plot also includes crystals that may significantly change their geometry to another local energy minimum. This figure shows that the Simple rates of the structures in the GA pool are predictive of their final rates after re-optimization, especially in the region where the rates are high.

GW+BSE

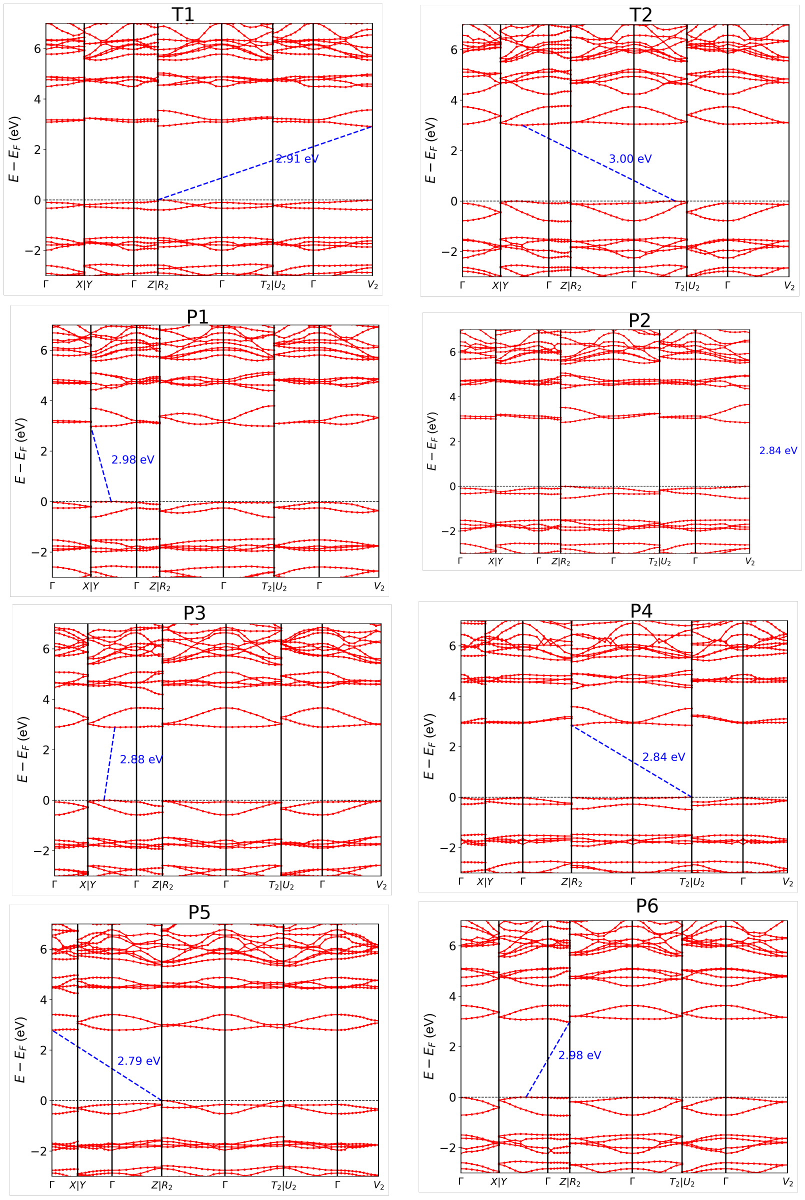

Figure S9: GW band structures of all the structures within 4 kJ/mol from the global minimum, including putative polymorphs and the known experimental forms, T1 and T2.

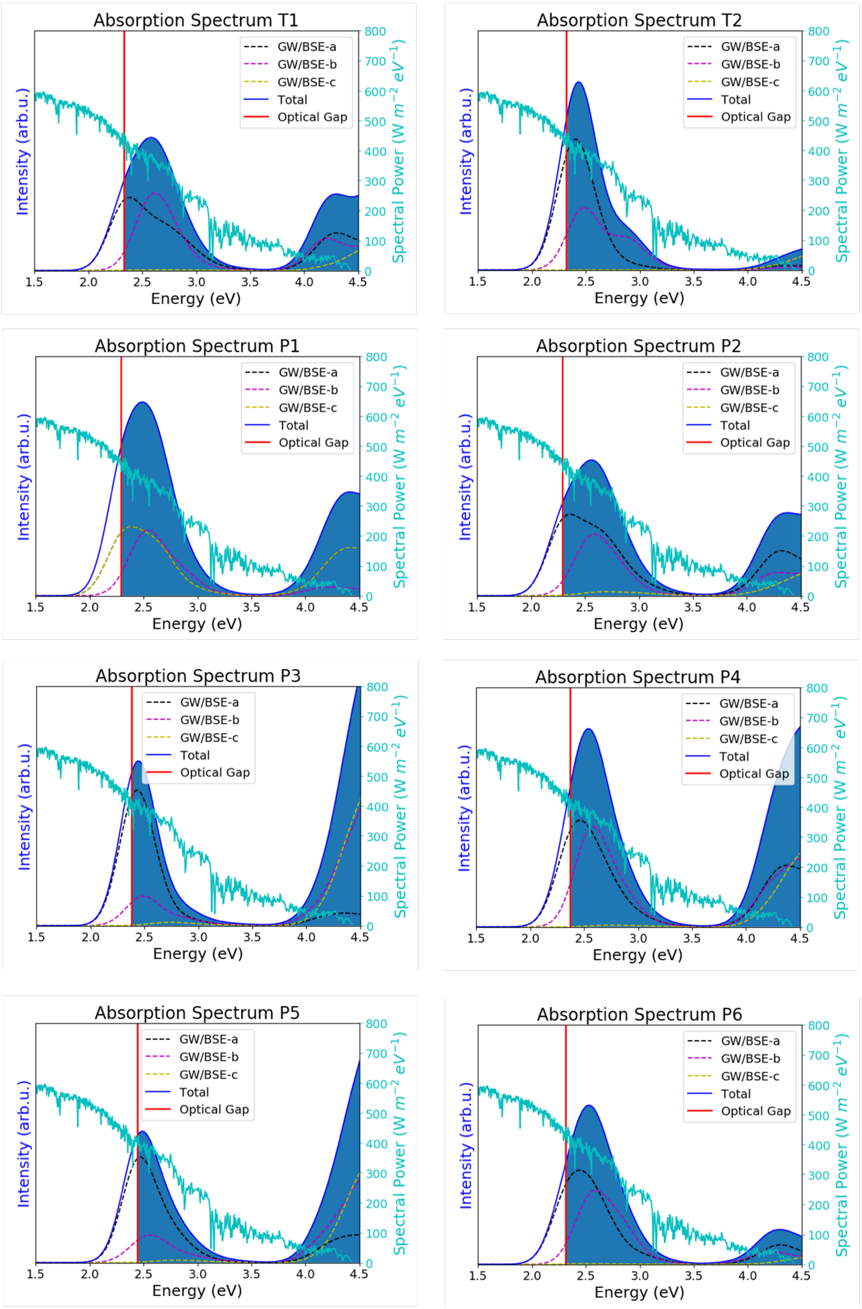

Figure S10: Absorption spectra, computed using BSE, of all the structures within 4 kJ/mol from the global minimum. The absorption spectra of the experimental forms, T1 and T2, are also shown.

Interaction chain Analysis

Interaction chain analysis was performed as described in Ref.[3] Periodic molecular chains were extracted from the T1 and T2 structures. Each periodic molecular chain only has one of the following type of interactions: face-to-face, side-to-face or edge-to-edge. The interaction energy, E_{inter}, of each periodic chain is defined as:

$$E_{inter} = \frac{E_{chain}}{Z} - E_{molecule} \quad (1)$$

where E_{chain} is the total energy of the extracted periodic chain, $E_{molecule}$ is the energy of the isolated molecule, and Z is the number of molecules in the unit cell of the interaction chain.

For the interaction chain analysis, the energies of the periodic interaction chains and the isolated molecule were evaluated by single point energy calculations with PBE+TS, PBE+MBD, and PBE0+MBD using *higher-level* settings. The k-points (k) in each direction were chosen to be the smallest integer that satisfies $k \times a \geq 25\mathring{A}$, where a is the lattice parameter in that direction. The geometries used to extract interaction chains for PBE+TS and PBE+MBD calculations were from units cells relaxed by each method respectively. The PBE0+MBD calculations used the same geometries as the PBE+MBD method, identical to the way calculations are carried out for the full crystal structures. The results are tabulated in Tables S1-S3.

The relative interaction energies, shown in Fig. S11 are averaged over all interaction chains of the same type and referenced to PBE0+MBD. Side-to-face and face-to-face interactions are present in both the T1 and T2 structures. Edge-to-edge interactions are only present in the T2 structure, along the c-axis. For all of these interactions, the interaction energy obtained with the PBE+TS method is significantly more negative than the interaction energy obtained with the PBE0+MBD method, which corresponds to over-stabilization. For side-to-face and face-to-face interactions, the interaction energies obtained with PBE+MBD are less negative than the interaction energies obtained with the PBE0+MBD, which cor-

responds to under-stabilization, whereas the edge-to-edge interactions are over-stabilized by PBE+MBD. We note that the lattice energies are not sums over the interaction energies. Whether a certain structure is under-stabilized or over-stabilized compared to another depends on the overall balance of different interactions.

Table S1: Energies of intermolecular side-to-face interactions computed using different DFT functionals and dispersion methods.

Structure	Direction	PBE+TS (kJ/mol)	PBE+MBD (kJ/mol)	PBE0+MBD (kJ/mol)
T1	$\langle 010 \rangle$	-52.1737	-42.9087	-44.5135
T1	$\langle 001 \rangle$	-25.0993	-20.6099	-21.6334
T1	$\langle 100 \rangle$	-86.1203	-69.3990	-72.9518
T2	$\langle 010 \rangle$	-53.7308	-44.5585	-46.0524
T2	$\langle 100 \rangle$	-86.8745	-70.5705	-73.7696

Table S2: Energies of intermolecular face-to-face interactions computed using different DFT functionals and dispersion methods.

Structure	Direction	PBE+TS (kJ/mol)	PBE+MBD (kJ/mol)	PBE0+MBD (kJ/mol)
T1	$\langle 100 \rangle$	-37.0896	-32.0837	-32.9126
T1	$\langle 110 \rangle$	-41.8321	-36.0472	-36.7408
T2	$\langle 100 \rangle$	-37.4252	-32.4211	-33.0779
T2	$\langle 110 \rangle$	-42.5960	-36.7667	-37.2682

Table S3: Energies of intermolecular edge-to-edge interactions computed using different DFT functionals and dispersion methods.

Structure	Direction	PBE+TS (kJ/mol)	PBE+MBD (kJ/mol)	PBE0+MBD (kJ/mol)
T2	$\langle 111 \rangle$	-56.0917	-49.3255	-49.3354
T2	$\langle 011 \rangle$	-13.0291	-11.9114	-11.2360
T2	$\langle 101 \rangle$	-47.9476	-42.4889	-42.5455
T2	$\langle 001 \rangle$	-8.5800	-7.6693	-7.0486

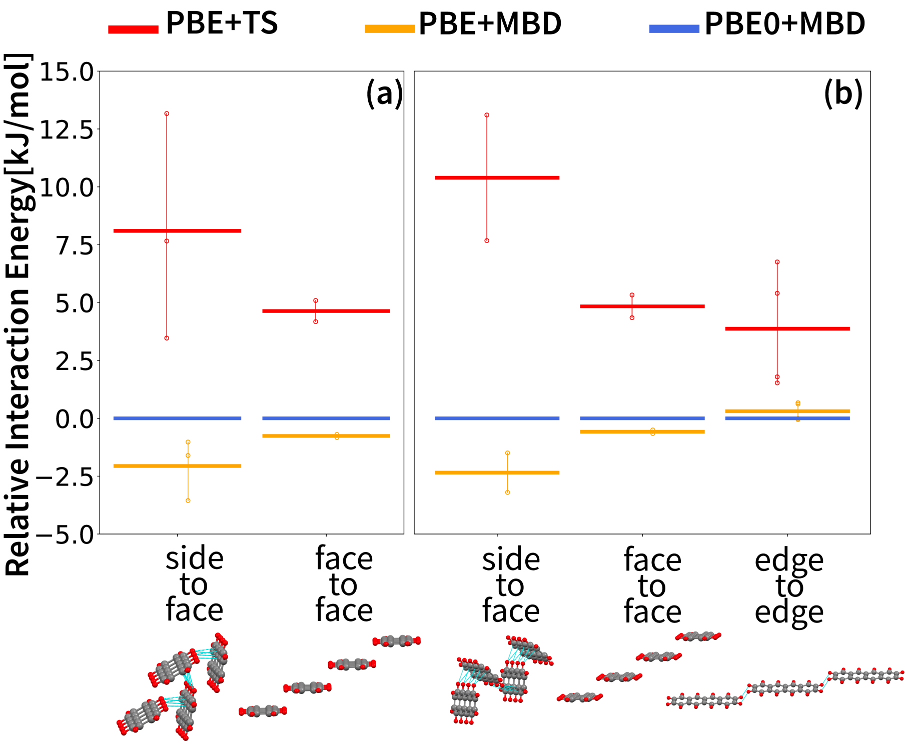

Figure S11: Interaction energy analysis of (a) T1 structure and (b) T2 structure for dominant interactions evaluated with PBE+TS (red), PBE+MBD (orange) and PBE0+MBD (blue). All interaction energies are shown relative to PBE0+MBD.

References

(1) Bier, I.; O'Connor, D.; Hsieh, Y.-T.; Wen, W.; Hiszpanski, A. M.; Han, T. Y.-J.; Marom, N. Crystal structure prediction of energetic materials and a twisted arene with Genarris and GAtor. *CrystEngComm* **2021**, *23*, 6023–6038.

(2) Tom, R.; Rose, T.; Bier, I.; O'Brien, H.; Vázquez-Mayagoitia, Á.; Marom, N. Genarris 2.0: A random structure generator for molecular crystals. *Comp. Phys. Comm.* **2020**, *250*.

(3) Bier, I.; O'Connor, D.; Hsieh, Y.-T.; Wen, W.; Hiszpanski, A. M.; Han, T. Y.-J.; Marom, N. Crystal structure prediction of energetic materials and a twisted arene with Genarris and GAtor. *CrystEngComm* **2021**, *23*, 6023–6038.
